# *Alpinia oxyphylla* Miq. and Its Active Compound *P*-Coumaric Acid Promote Brain-Derived Neurotrophic Factor Signaling for Inducing Hippocampal Neurogenesis and Improving Post-cerebral Ischemic Spatial Cognitive Functions

**DOI:** 10.3389/fcell.2020.577790

**Published:** 2021-01-18

**Authors:** Yacong He, Shuang Chen, Bun Tsoi, Shuhua Qi, Bing Gu, Zhenxing Wang, Cheng Peng, Jiangang Shen

**Affiliations:** ^1^School of Chinese Medicine, Li Ka Shing Faculty of Medicine, The University of Hong Kong, Hong Kong, China; ^2^Medical Technology School, Xuzhou Key Laboratory of Laboratory Diagnostics, Xuzhou Medical University, Xuzhou, China; ^3^Chengdu University of Traditional Chinese Medicine, Chengdu, China; ^4^Key Laboratory of Standardization of Chinese Herbal Medicines of Ministry of Education, Pharmacy College, Chengdu University of Traditional Chinese Medicine, Chengdu, China

**Keywords:** *Alpinia oxyphylla* Miq., *p*-coumaric acid, brain-derived neurotrophic factor, hippocampal neurogenesis, ischemic stroke

## Abstract

Alpinia *oxyphylla* Miq. (AOM) is a medicinal herb for improving cognitive functions in traditional Chinese medicine for poststroke treatment, but its efficacies and underlying mechanisms remain unknown. In the present study, we tested the hypothesis that AOM could induce adult hippocampal neurogenesis and improve poststroke cognitive impairment *via* inducing brain-derived neurotrophic factor (BDNF) signaling pathway. In order to test the hypothesis, we performed both *in vivo* rat experiments using transient middle cerebral artery occlusion (MCAO) model and *in vitro* neural stem cell (NSC) experiments using oxygen–glucose deprivation plus reoxygenation. First, AOM treatment significantly up-regulated the expression of BDNF, tropomycin receptor kinase B (TrkB), and phosphorylated AKT (p-AKT) in the hippocampus, enhanced adult hippocampal neurogenesis, and improved the spatial learning/memory and cognitive functions in the post-MCAO ischemic rats *in vivo*. Next, *in vitro* studies confirmed *p*-coumaric acid (P-CA) to be the most effective compound identified from AOM extract with the properties of activating BDNF/TrkB/AKT signaling pathway and promoting NSC proliferation. Cotreatment of BDNF/TrkB-specific inhibitor ANA12 abolished the effects of P-CA on inducing BDNF/TrkB/AKT activation and the NSC proliferation. Finally, animal experiments showed that P-CA treatment enhanced the neuronal proliferation and differentiation in the hippocampus, improved spatial learning and memory functions, and reduced anxiety in the transient MCAO ischemic rats. In conclusion, P-CA is a representative compound from AOM for its bioactivities of activating BDNF/TrkB/AKT signaling pathway, promoting hippocampal neurogenesis, improving cognitive functions, and reducing anxiety in post–ischemic stroke rats.

## Background

Stroke is a major disease burden with high mortality and disability caused by impeded blood flow to the brain (Johnston et al., 2018). Stroke victims suffer from motor dysfunctions, paralysis, cognitive impairment (Mijajlović et al., 2017), and even sudden death (Donnan et al., 2008). Current therapeutic strategies including thrombolysis with tissue plasminogen activator and mechanical thrombectomy are effective for the recanalization of blood flow to the ischemic brain but have restrictive therapeutic windows within 4.5 and 6 h, respectively. The delayed thrombolytic treatment beyond the golden therapeutic windows carries the risks of the complications of intracranial hemorrhagic transformation and increases the mortality of stroke patients (Gravanis and Tsirka, 2008). To date, there is no medication for neuroprotection and neurogenesis in stroke treatment.

Adult neurogenesis brings new hope for seeking therapeutic targets to repairing a damaged brain and improving functional recovery in poststroke patients (Wang et al., 2012; Sun et al., 2013). The subventricular zone (SVZ), lining the lateral ventricle, and the subgranular zone in the dentate gyrus (DG) are the central regions of adult neurogenesis in the brain (Lois and Alvarez-Buylla, 1994; Eriksson et al., 1998). The newborn neurons in DG contribute to adult hippocampal neurogenesis for improving learning and memory functions (Deng et al., 2010). Adult hippocampal neurogenesis includes a series of sequential events to generate new excitatory granule cells in the DG. The newborn neurons go through several consecutive generation stages before integrating into the hippocampal circuit. First, radial glia-like precursor cells (type 1 cells) with astrocytic properties express the biomarkers of neural stem cells (NSCs). The cells give rise to intermediate progenitor cells with first glial (type 2a) and then neuronal (type 2b) phenotype. After developing into a migratory neuroblast-like stage (type 3), these newborn lineage-committed cells enter a maturation stage and extend their dendrites into the molecular layer and their axon to CA3, eventually forming granule cells (Markakis and Gage, 1999). By labeling various biomarkers, we can track the procedures of hippocampal neurogenesis at different stages. For example, doublecortin (DCX) shows a complete overlap in expression with the polysialylated neuronal cell adhesion molecule in the hippocampus, which are used as surrogate markers for adult neurogenesis (Kempermann et al., 2015). DCX is expressed at the stages of proliferation to postmitotic maturation and commonly used as a neurogenic biomarker (Encinas and Enikolopov, 2008; Kempermann et al., 2015; Chen et al., 2020). DCX and neuronal nuclei (NeuN) are commonly used as neurogenic biomarkers and costained with exogenous cell tracer 5′-bromo-2′-deoxyuridine (BrdU) to identify newly formed neurons and indicate adult neurogenesis in the central nervous system. BrdU, a thymidine analog, can be transferred into dividing cells by DNA synthesis (Lehner et al., 2011). After integrating into the new DNA, BrdU will be passed to daughter cells (Kee et al., 2002). The proliferation and differentiation of NSCs and/or neural progenitor cells (NPCs) can be marked by costaining BrdU and DCX and/or NeuN to identify immature or mature neurons, respectively (Wojtowicz and Kee, 2006; Gao et al., 2018). Thus, these biomarkers facilitate the studies on exploring the underlying mechanisms of adult neurogenesis and seeking drug candidates for functional recovery in poststroke treatment.

Many extrinsic and intrinsic factors, which are generated from NSCs/NPCs, neurogenic niche and microenvironment form complex network regulations for adult neurogenesis in postischemic brain. Notch signaling, Wnt/β-catenin signaling pathway, sonic Hedgehog pathway, bone morphogenetic proteins, growth factors, neurotrophic factors, and neurotransmitters are well recognized as cellular signaling cascades contributing to poststroke neurogenesis (Bond et al., 2012; Faigle and Song, 2013; Liu et al., 2018; Wagenaar et al., 2018; Ho et al., 2020; Xu et al., 2020). Among them, brain-derived neurotrophic factor (BDNF) is a critical neurotrophic factor participating in the regulations of proliferation, differentiation, survival, and maturation of NSCs for neurogenesis. BDNF is formed by the combination of signal peptide, prodomain, and mature BDNF precursor form. The prodomain of BDNF is removed by cell proteases to promote the secretion of mature BDNF. By binding to tropomycin receptor kinase B (TrkB) receptor, BDNF phosphorylation activates downstream pathways, including MAP kinase/CREB, PI-3 kinase/Akt, and Ras/Raf/MEK/Erk signaling pathways (Mohammadi et al., 2018). BDNF and its receptor TrkB are highly expressed in hippocampal region for memory formation (Egan et al., 2003). BDNF promotes neurological functional recovery in neonatal hypoxic–ischemic brain injury (Im et al., 2010). Endothelial-derived BDNF formation promotes the vasculature-mediated migration of neuronal precursors in the ischemic striatum (Grade et al., 2013). Overexpression of BDNF increases the survival rates of the transplanted NSCs and promotes neurological functional recovery in experimental ischemic stroke animal models (Lee et al., 2010; Chang et al., 2013; Rosenblum et al., 2015). Adenoviral transduction of BDNF gene in human bone marrow–derived mesenchymal stem cells (MSCs) efficiently promotes its survival after transplantation, enhances the proliferation of endogenous NSCs, and promotes functional recovery in a middle cerebral artery occlusion (MCAO) rat model (Jeong et al., 2014). BDNF increases the survival and differentiation of dental pulp stem cells (DPSCs) and promotes the recovery of neurological functions in a cerebral ischemia animal model with DPSC transplantation (Zhang X. et al., 2018). In addition, plasma BDNF level is used as a biomarker to reflect cell viability and functional recovery in the transplantation of MSCs (Nakamura et al., 2019). On the contrary, antisense oligonucleotides of BDNF inhibit proliferation and differentiation of NSCs in the ischemic brains (Li et al., 2017). Suppressing adult hippocampal neurogenesis worsens cognitive performance and decreases preexisting dentate neurons. Combined mimic exercise and elevating BDNF levels to enhance adult neurogenesis improve cognitive function and protect against subsequent neuronal cell death in an Alzheimer disease (AD) mouse model (Choi et al., 2018). Therefore, BDNF is an important therapeutic target for adult neurogenesis and functional recovery in poststroke treatment.

Traditional Chinese medicine (TCM) has been used for stroke treatment for centuries in China and East Asia. The direct experiences from human subjects provide fast-track sources for drug discovery. *Alpinia oxyphylla* Miq. (AOM) is a commonly used medicinal plant for improving cognitive functions in TCM formulas, but its efficacies and scientific basis remain unclear. In recent studies, AOM treatment has revealed to attenuate neuronal cell death, improve β-amyloid–induced cognitive impairment, reduce neuronal abnormalities in the cortex and hippocampus, and enhance memory in mice (Shi et al., 2014, 2015). AOM exerts antidepressant-like effects by targeting TrkB receptor–mediated pERK/pCREB/BDNF signal systems (Yan et al., 2016). However, whether AOM has neurogenesis-promoting effects for the functional recovery of learning and memory in ischemic stroke remains unknown. Herein, we report that AOM could promote hippocampal neurogenesis and enhance the functional recovery of learning and memory by inducing BDNF signaling pathway in transient ischemic stroke treatment. Furthermore, we have identified *p*-coumaric acid (P-CA) as the representative compound from AOM with the properties of promoting BDNF signaling for inducing hippocampal neurogenesis and improving functional recovery in post–ischemic stroke rat model.

## Materials and Methods

### Herbal Materials and Reagents

*Alpinia oxyphylla* Miq. was purchased from Hong Kong Huaxin Pharmaceutical Inc. Ltd. (serial no. 170807), which was originally from Hainan province, China. Mouse multipotent neural progenitor or stem-like cells C17.2 [07062902, European Collection of Authenticated Cell Cultures (ECACC)] were purchased from Sigma–Aldrich (St. Louis, MO, United States). Thymidine analog BrdU (ab142567) and antibodies for BrdU (ab6326), SOX2 (ab97959), and BDNF (3B2) were purchased from Abcam (Cambridge, United Kingdom). Other antibodies for Ki67 (D3B5), NeuN (D4G4O), DCX (A8L1U), phospho-AKT (Ser473), AKT (C67E7), β-actin (8H10D10), and GAPDH (5174) were obtained from Cell Signaling Technology (Danvers, MA, United States). TrkB (sc-12) was ordered from Santa Cruz (Dallas, TX, United States). ANA12 was provided from Cayman Chemical (Ann Arbor, MI, United States). Solvents for high-performance liquid chromatography (HPLC) and liquid chromatography–mass spectrometry (LC-MS) analysis were at HPLC-grade. Compounds for quality control analysis including kaempferol, 5-hydroxymethylfurfural, chrysin, P-CA, nootkatone, (−)-epicatechol, catechin, protocatechuic acid, protocatechuic aldehyde, and tectochrysin with purity of greater than 98% were purchased from Chengdu Push Bio-technology Co., Ltd., China.

### AOM Extraction and Drug Solution

Dry raw materials of AOM (2 kg) were grinded into scraps and soaked in 20 L of 70% ethanol overnight. The mixture was sonicated three times (40 min per time). The solution was concentrated by rotary evaporator at 60°C. After ethanol was evaporated, the solution was freeze-dried to obtain AOM extraction. For qualitative and quantitative analyses, we dissolved AOM extraction in ethanol (10 mg/mL) by ultrasonic for 30 min and performed HPLC analysis and LC-MS analysis.

In the LC-MS analysis, by comparing the ESI-MS data from QDa positive/negative scanning of AOM extract (Supplementary Figure 1) and the reported ESI-MS data of the potential neuronal bioactive components in AOM (Zhang Q. et al., 2018), we speculated 14 compounds in the AOM extracts, and we collected those compounds (>98% purity) for further verifications of the compounds in the AOM extract. Stock solution of each compound was made by dissolving 0.2 mg in 2 mL of ethanol under ultrasonic for 30 min. 100 μL of stock solution was taken to obtain mixed stander solution. With HPLC and LC-MS experiments, 10 compounds were identified in the AOM extract, including 5-hydroxymethylfurfural, protocatechuic acid, catechin, protocatechuic aldehyde, (−)-epicatechol, P-CA, kaempferol, chrysin, nootkatone, and tectochrysin. After P-CA was identified to be the most effective compound, we then prepared the test solution by dissolving 50 mg of P-CA into 50 mL of 0.1% phosphoric acid–water and methanol (70:30) under ultrasonic for 30 min.

### Quality Control Analysis

We performed quality control study on AOM extract and characterized the chemical profiles of the AOM solution by using HPLC. Chromatographic separations were operated on an Agilent 1100 Series HPLC system with Shimadzu C18 column (4.6 × 250 mm, 5 μm). The following chromatographic parameters were used in the study: mobile phase A (acetonitrile) and mobile phase B (0.1% trifluoroacetate–water), flow rate at 1 mL/min, column temperature at 30°C, and detection wavelength at 220 nm. The gradient profiles were from 0 to 60 min, and the percentage of mobile phase A gradient was increased from 5 to 100%. LC-MS was operated by using Waters LC/MS ACQUITY QDA with CORTECS C18 column (4.6×50 mm, 2.7 μm). LC-MS was measured under the condition of ionization method-ESI (+/−), with a scanning range from 100 to 1,000. The following chromatographic parameters were used: mobile phase A (0.1% formic acid water) and mobile phase B (0.1% formic acid acetonitrile), flow rate at 0.5mL/min, column temperature at 30°C, and detection wavelength at 220 and 254 nm. The gradient profile was that mobile phase B was increased from 5 to 95% for 6 min and held 95% for 3 min.

### Quantitative Analysis

We selected P-CA as a marker compound for quantitative control of the AOM extraction. P-CA was determined at the wavelength of 309 nm by using Shimadzu LC-2030c HPLC system and Waters symmetry C18 (4.6 × 150 mm, 5 μm). The chromatographic analysis was operated at the parameters: flow rate at 1 mL/min, 10-μL sample injection volume, column temperature at 35°C, detection wavelength at 309 nm, mobile phase A (0.1% phosphoric acid–water), and mobile phase B (Methanol), constantly running 60 min. In quantitative experiments, linearity, precision, accuracy, sensitivity, repetitive, and stability were detected. The limits of detection (LODs) and limits of quantitation (LOQs) under the present conditions were determined at a signal-to-noise ratio of approximately 3 and 10, respectively.

To obtain calibration curves, 10 concentrations of P-CA were analyzed in HPLC. The test solutions were made into six replicates in parallel manner for the precision of the measurement results. One test solution was injected for six times constantly to examine the repetition of the HPLC system. The accuracy was evaluated by the recovery rate test. In the verification study, equal amount of mixed sample solution and reference substance spiked solution was mixed and allocated into six solutions for recovery rate test. The actual value was compared with the theoretical value to calculate the recovery rates. The stability of test solution and reference solution during storage was measured by testing the changes of the main peak area after the test solution and the reference solution were placed at room temperature for 0, 2, 4, 6, 8, 12, and 18 h, respectively.

### Animals and Experimental Groups for AOM Extraction and P-CA Study

Male Sprague–Dawley (S.D.) rats (12 weeks old, weighing 260–290 g) were obtained from Laboratory Animal Unit, The University of Hong Kong. All animals were kept with tap water and standard food pallets, within a stable environment (temperature 25°C ± 2°C, humidity 40%, 12/12-h dark/night cycle) in an animal room. All animal experimental procedures and care were approved by the University Committee on the Use of Live Animals in Teaching and Research (CULATR no. 4664-18). The rats were randomly divided into three groups before surgery: sham-operated group, MCAO model group, and MCAO plus AOM treatment group. For P-CA study, three concentrations of P-CA were designed (low 50 mg/kg, middle 100 mg/kg, high 200 mg/kg treatment groups).

### Surgical Protocol

Sprague–Dawley rats were subjected to MCAO to induce cerebral ischemia animal model. The rats were anesthetized with 4% isoflurane and maintained at 2% isoflurane. The rats were then placed on a warm pat to maintain body temperature. Neck hair was shaved out with shaving cream, and the neck skin was disinfected three times with iodine and 70% ethanol. Under a surgical microscope, a neck midline incision was made, and the superficial fascia was incised. Under the superficial fascia, a careful blunt dissection was performed to expose the common carotid artery (CCA), external carotid artery (ECA), and internal carotid artery (ICA) without damaging the vagus nerve. A 6-0 nylon suture was used to tighten the CCA and ECA. The ECA was cut and stumped with a needle. A 0.36-mm nylon suture covered with silicone was carefully inserted and pushed into the ICA until reached to the left middle cerebral artery in the place with the feeling of slight resistance. The ICA and ECA were carefully tightened with 6-0 nylon suture. The incision was closed with 3-0 nylon sutures. The muscle layer was sutured with a 6-0 polyglactin suture, and the incision layer was sutured with a 3-0 nylon suture. After 2 h of MCAO ischemia, the intraluminal suture was removed to induce reperfusion. The rats were kept warm until recovery from anesthesia. After that, we monitored the well-being of the rats, which were presumably free to access water and food.

### Drug Preparation and Administration

**FIGURE 1:**
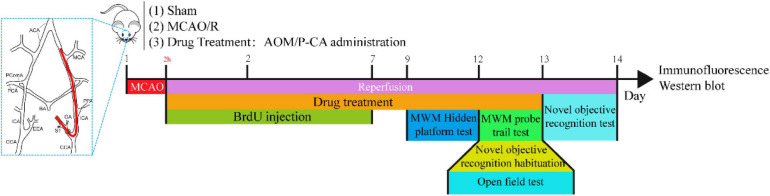
Schematic illustration of experiment protocol for MCAO cerebral ischemia–reperfusion. Male Sprague–Dawley (S.D.) rats were subjected to 2 h of MCAO cerebral ischemia plus 14 days of reperfusion. AOM extract (6.3 g/kg, dissolved in 5% ethanol and 5% PEG 400), P-CA (50, 100, 200 mg/kg, dissolved in saline), and vehicle solution were orally given to the rats at onset of reperfusion after 2 h of MCAO cerebral ischemia and daily administered to the rats until day 13 of reperfusion. BrdU (50 mg/kg) was intraperitoneally injected to all animals for 7 consecutive days. Behavioral tests were conducted from day 9 to day 14.

The *in vivo* animal experiment protocol is shown in Figure 1. The AOM extraction was dissolved in a water solution with 5% ethanol plus 5% polyethylene glycol 400 (PEG 400). In the AOM treatment group, at the onset of reperfusion, the AOM extraction was orally administered into the rats at the dosage of 6.3 g/kg, equivalent to the daily dose of raw herbal material in human subject. The AOM extraction was given to the rats for 13 consecutive days. In the P-CA treatment group, different dosages of P-CA (dissolved in saline at 50, 100, and 200 mg/kg) were intragastrically administered into the rats for 13 consecutive days. Sham-operated group and MCAO model group were intragastrically administered with 1 mL of vehicle solution. Body weight was daily recorded after surgery. The brain samples were collected at day 14 after cerebral ischemia for immunofluorescence staining study and Western blot.

### *In vivo* BrdU Incorporation

BrdU was intraperitoneally injected into the rats for 7 consecutive days at the dosage of 50 mg/kg according to that previously described (Wojtowicz and Kee, 2006).

### Behavioral Test for Neurological Severity Score Assessment

Neurological deficit was examined by using the modified neurological severity score (mNSS). The mNSS score reflects the neurological functions related to motor (muscle status, abnormal movement), sensory systems (visual, tactile, and proprioceptive), and the reflex and balance capabilities. The criteria of the mNSS scores ranging from 0 to 18 (0 for accessible/normal, 18 for functional maximum lesion) were adopted according to previous report (Chen et al., 2001).

### Morris Water Maze Test

Morris water maze test was performed to assess the cognitive functions in the sham control group, MCAO vehicle group, MCAO plus AOM treatment group, and MCAO plus P-CA treatment groups. The hidden platform training was carried out on the ninth day after surgery. In 4 days of training, each rat was allowed 60 s to find the platform in water tank twice per day. The probe trial was carried out on the 13th day in the whole treatment period. The test was recorded with a time limit of 70 s (Su et al., 2017). Latency time of reaching the platform, the distance, and the swimming time in the target region of the probe tail test were recorded for testing spatial learning and memory functions.

### Open-Field Test

The open-field test was performed at the 13th day by using an 80 × 80 × 50-cm acrylic black box. The square was divided into the center and outer edge. At the starting point, the rats were placed into the center square of the open field. Each 10-min trial was videotaped by an overhead camera. Distance, time, entries time in center, and total distance in the whole filed were recorded by an overhead video and analyzed by using Smart 3.0 system (RWD, Delaware, United States).

### Novel Object Recognition Test

Novel objective recognition habituation was performed at the 13th day after surgical operation. Rats were placed into an 80 × 80 × 50-cm acrylic black box for 10 min. In the test, rats were first placed in the open box at the sample stage, allowed to explore two identical objects, and then returned to their cages. With a delay of 60 min, at the testing phase, the rats were returned to the box where they were exposed to two different objects: one was the same as the previously encountered objects, and the other was a novel object for the rats. The total time of exploration to those objects was 20 s (Lueptow, 2017). The exploration time on the novel object in the test phase was examined.

### Cell Culture

Mouse multipotent neural progenitor or cerebellum stem-like C17.2 cells were originally from ECACC. The cells were maintained in 75-cm^2^ vented culture flasks using high-glucose Dulbecco modified eagle medium (DMEM, Gibco) with 10% heat-inactivated fetal bovine serum (Gibco), 1% penicillin/streptomycin (Gibco), and 1% 2 mM L-glutamine (Gibco). The C17.2 cells were cultured in a 37°C humidified incubator with a 5% CO2/95% air atmosphere.

### Oxygen–Glucose Deprivation Plus Reoxygenation Model and Drug Treatment

To mimic ischemic/reperfusion condition *in vitro*, C17.2 cells were exposed to the oxygen–glucose deprivation plus reoxygenation (OGD/R) condition. To induce OGD, these cells were cultured with glucose-free DMEM (Gibco) and incubated in a 37°C CO_2_ incubator (Eppendorf New Brunswick Galaxy 48R) with oxygen control as 0.1% O_2_ and 5% CO2/94.9% N_2_. The real-time condition was monitored by incubator reading. The C17.2 cells were exposed to the OGD for a 4-h period and following 20 h of reoxygenation by the replacement of DMEM with high glucose and returned to a 37°C humidified incubator with a 5% CO2/95% air atmosphere. The C17.2 cells were treated and incubated with corresponding compounds for 20 h after OGD.

To identify the bioactive compounds contributing to the neurogenic effects of AOM extract, we examined the effects of 10 identified compounds on inducing BDNF/TrkB/AKT expression and promoting proliferation in the C17.2 cells under OGD and reoxygenation conditions. These compounds, including 5-hydroxymethylfurfural, protocatechuic acid, catechin, protocatechuic aldehyde, (−)-epicatechol, P-CA, kaempferol, chrysin, nootkatone, and tectochrysin, were dissolved in dimethyl sulfoxide to obtain 100 mM stock solutions, which were added into the cells with final concentrations of 1, 10, and 100 μM. The cells were seeded in the 24- well plates with poly-D-lysine–coated (Trevigen, Gaithersburg, MD, United States) cover slips and normal 96-well plates, 6-well plates at a density of 2.5 × 10^4^ cells/mL. The cells were allocated into the groups of control, OGD, and the OGD plus the selected compounds with three dosages.

### *In vitro* BrdU Labeling

BrdU stock solution (10 mM) was prepared and diluted into 10 μM BrdU labeling solution with cell culture medium. The BrdU labeling solution was filtered through a 0.2-μm filter under sterile condition. After 20 h of reoxygenation, the treatment medium in 96-well plates was replaced with the BrdU labeling solution for 1 h in 37°C and 5% CO2 incubator. After that, the BrdU labeling solution was removed, and the cells were washed twice in phosphate-buffered saline (PBS).

### Immunofluorescence

For *in vivo* animal experiments, postfixed brain tissues were immersed in 30% sucrose solution at 4°C for complete dehydration, embedded in O.C.T., and cut into 30-μm sections. The frozen slices were retrieved with sodium citrate buffer. For *in vitro* cell experiments, after labeling with BrdU for 1 h, the cells were fixed with 4% paraformaldehyde for 15 min. The retrieved sections and the cells were incubated with 2 M HCl under room temperature for 1 h and then washed with 1% PBST (PBS + 1% Triton) three times for 15 min. Afterward, cells were blocked with 5% goat serum (GS) in 3‰ PBST (PBS + 3‰ triton) for 1 h. Sections were stained with primary antibodies including BrdU (rat, 1:400), Ki67 (rabbit, 1:400), DCX (rabbit, 1:400), and NeuN (rabbit, 1:400) overnight. The C17.2 cells were costained with primary antibodies BrdU (rat, 1:400), Ki67 (rabbit, 1:400), and SOX2 (rabbit, 1:400). After incubation with primary antibodies at 4°C overnight, the samples were washed with 3‰ PBST three times for 15 min. All slices were stained with anti-rat 488 (1:800) and anti-rabbit 568 (1:800) for 2 h and counterstained the nucleus with DAPI (4′,6′-diamidino-2-phenylindole) for 15 min in room temperature. Brain sections and cell samples were mounted with fluorescence mounting medium (Dako Agilent, Santa Clara, CA, United States), whereas the cells for high content screening (HCS) experiments were kept in PBS. Immunofluorescent images were captured by Carl Zeiss LSM 800 Confocal Laser Scanning Microscope and analyzed by ZEN offline. HCS experiment was performed by using GE Healthcare Life Sciences IN Cell Analyzer 6500HS and analyzed by IN Carta offline.

### Western Blot Analysis

Proteins were extracted by radioimmunoprecipitation assay buffer with 1% protease and phosphatase inhibitor cocktails (CST). The protein concentration was detected by BCA Protein Assay Kit (Thermo Fisher Scientific, United States). Protein lysates were separated by electrophoresis with 11% sodium dodecyl sulfate polyacrylamide (sodium dodecyl sulfate–polyacrylamide gel electrophoresis) gel, transferred to polyvinylidene fluoride membrane, and immunoblotted with primary antibodies including BDNF (Abcam, mouse, 1:1,000), TrkB (Santa Cruz, mouse, 1:100), AKT (CST, rabbit, 1:1,000), p-AKT (CST, rabbit, 1:1,000), β-actin (CST, mouse, 1:5,000), and GAPDH (CST, rabbit, 1:5,000) separately. Followed by horseradish peroxidase–conjugated secondary antibodies (1:2,000) incubation. Signals were detected by chemiluminescent ECL Select Kit (GE Healthcare, Chicago, IL, United States), captured by the Gel-Doc system (Bio-Rad, Hercules, CA, United States), and analyzed by Image Lab software (Bio-Rad, Hercules, CA, United States).

### Statistical Analysis

Data analysis was performed by using Prism 8 (GraphPad Software, United States). All data were expressed as mean ± SEM. Student *t*-tests were used for two groups of designed experiments, and two-way analysis of variance was used to analyze multiple groups of comparisons followed by Tukey multiple-comparisons test to determine the difference between two groups where appropriate. Statistical significance was defined as *p* < 0.05.

## Results

### Quality Control Study of AOM Extraction

**FIGURE 2:**
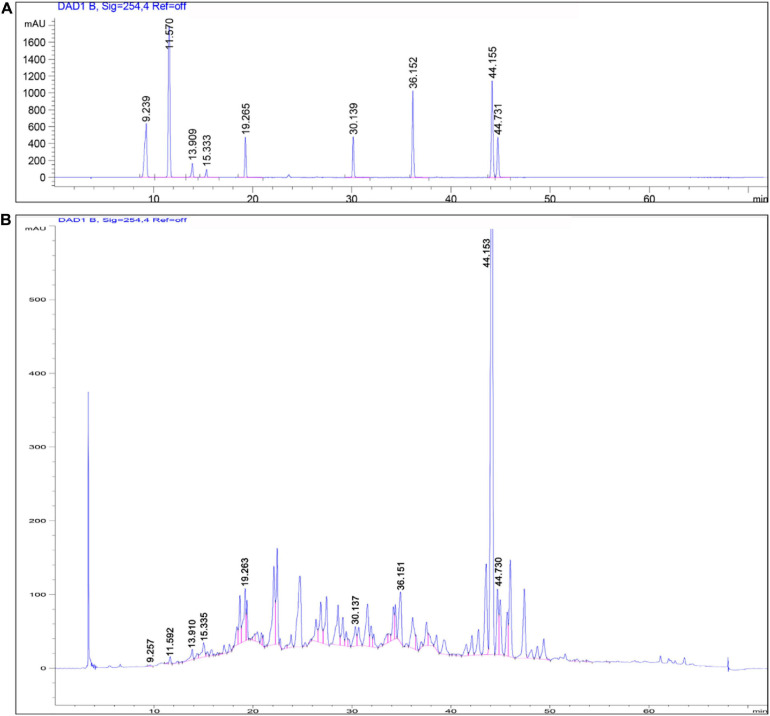
HPLC chromatograms of mixed chemical standards **(A)** and AOM extract **(B)**. Parameters for HPLC detection: Shimadzu C18 column (4.6 × 250 mm, 5 μm); mobile phase: gradient profile with acetonitrile (ACN)—0.1% trifluoroacetate–water from 5 to 100% in 60 min. Column temperature: 30°C; flow rate: 1.0 mL/min; UV detection: 220 nm.

**FIGURE 3:**
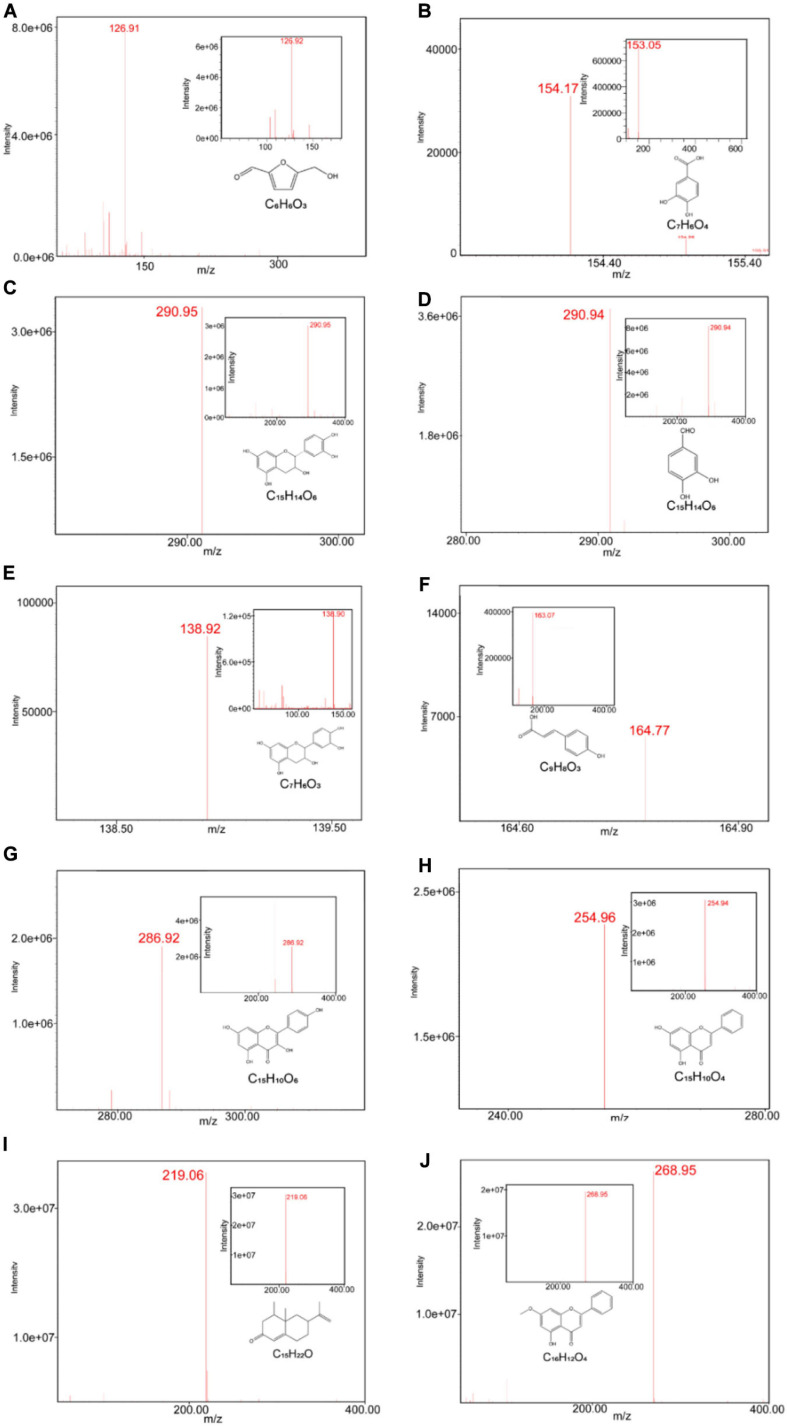
ESI-MS chromatograms of AOM extract. LC-MS was operated on Waters LC/MS ACQUITY QDA with CORTECS C18 column (2.7 μm 4.6 × 50 mm), measured under condition of ionization method-ESI (+/−) and scanned range from 100 to 1,000. ESI-MS chromatograms of 5-hydroxymethylfurfural **(A)**, protocatechuic acid **(B)**, catechin **(C)**, (−)-epicatechol **(D)**, protocatechuic aldehyde **(E)**, *p*-coumaric acid **(F)**, kaempferol **(G)**, chrysin **(H)**, nootkatone **(I)**, tectochrysin **(J)**, and their standards.

We first conducted quality control study to identify the chemical ingredients of AOM extract using HPLC. We optimized the chromatographic conditions and obtained a well-separated chromatogram (Figure 2). We compared the retention time of AOM extract and mixed standards. The structures and retention time of those compounds are summarized in Table 1. By combining the results in LC-MS (Figure 3 and Supplementary Table 1), we confirmed 10 compounds in AOM extract, including 5-hydroxymethylfurfural, protocatechuic acid, catechin, protocatechuic aldehyde, (−)-epicatechol, P-CA, kaempferol, chrysin, nootkatone, and tectochrysin. These identified compounds can be catalog as five flavonoids, three phenolic acid, one eremophilane, and one other compound.

**TABLE 1:** Representative compounds identified in AOM extract.

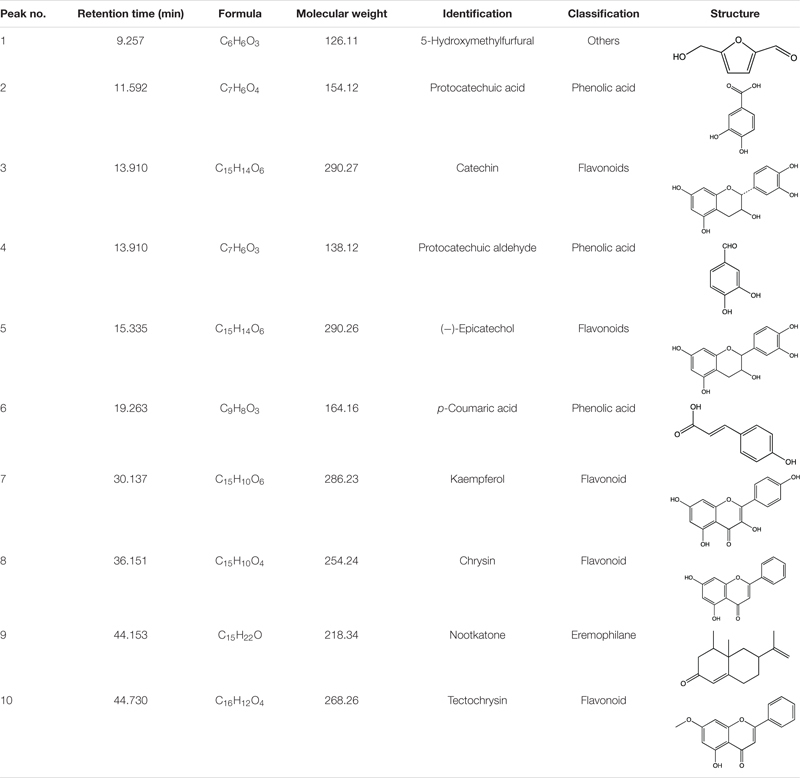

We then established an HPLC method to quantitatively analyze P-CA as a marker compound in AOM extract. We examined the linearity, repetition, precision, accuracy, and stability of the HPLC method for P-CA detection. The standard curve was y = 7.5275×10^7^x−1.5917×10^4^ with correlation coefficients (*r*) of 0.9998. The LODs and LOQs were 0.003163 and 0.01265 μg/mL, respectively. The relative standard deviation (RSD) of the test solution was 3.41% for the repetitive and 1.75% for the precision results. The accuracy was assessed by overall recovery rate, which was 90% to 108%, and the RSD was 0.39%. The RSD of the stability assay for the test solution within 18 h was 4.15%, and the RSD rates of the interday precision and intraday precision were 2.48% and 2.06%, respectively. These results indicate that the HPLC method was highly sensitive and reliable, and the concentration of P-CA was identified to be 4.6 μg/g in AOM extract.

### AOM Treatment Increases Body Weight and Promotes Spatial Learning and Memory in Post-MCAO Ischemic Rats

**FIGURE 4:**
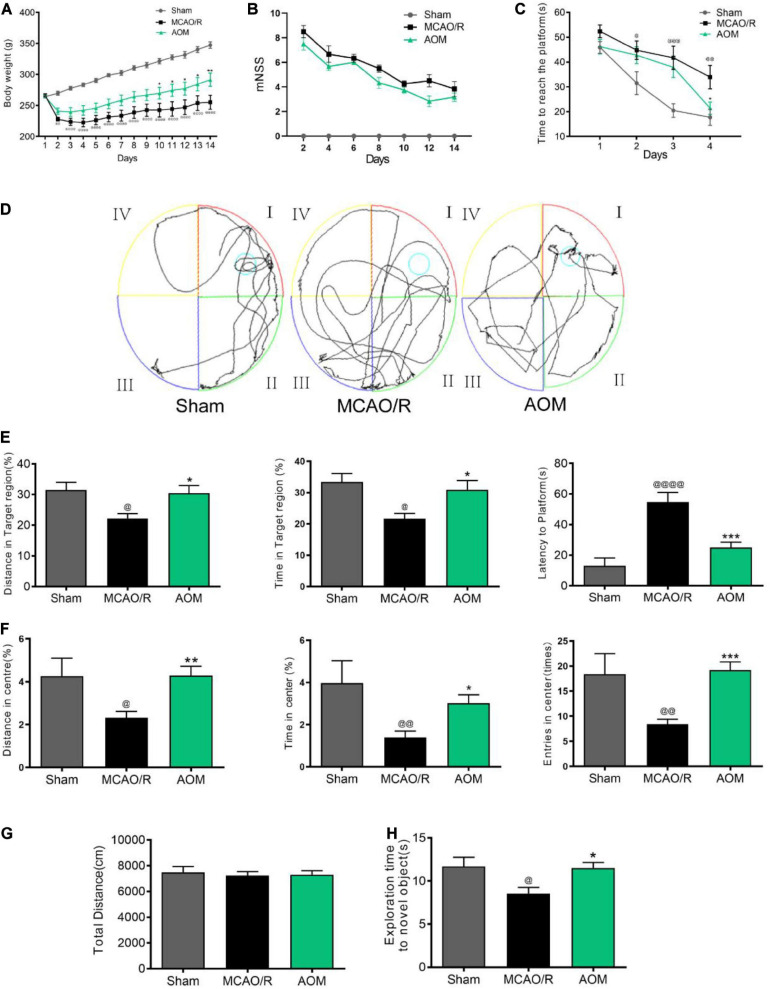
AOM increased body weight and improved spatial learning and memory capacity in transient MCAO rats. S.D. rats were divided into sham control group (Sham), MCAO cerebral ischemia–reperfusion vehicle group (MCAO/R), and MCAO/R plus AOM treatment group (AOM). The rats were subjected to 2 h of MCAO cerebral ischemia plus 14 days of reperfusion. AOM extract (6.3 g/kg, dissolved in 5% ethanol and 5% PEG400) and vehicle solution were orally given to the rats at onset of reperfusion after 2 h of MCAO cerebral ischemia and daily administered for 13 days of reperfusion. **(A)** Body weight changes in sham control, MCAO/R, and AOM groups; **(B)** mNSS in sham control, MCAO/R, and AOM groups. The mNSS was assessed every 2 days. **(C–E)** Morris water maze test for evaluating spatial learning and long-term memory in transient MCAO rats: **(C)** Escape latency in 4 days of hidden platform test. **(D)** Swimming routes in the probe trail test. **(E)** Distance and time in the target quadrant of the probe trial and latency time before reaching platform region. **(F,G)** Open-field test was used to measure anxiety in transient MCAO rats. Distance, time, and entries in center region were recorded in the sham control, MCAO/R, and AOM treatment groups. AOM treatment reduced the anxiety **(F)** but had no effect on total distance moved during habituation **(G)**. **(H)** Novel objective recognition test for short-term memory: AOM increased exploration time on novel object in test phase of the MCAO rats. Data were presented as mean ± SEM (*n* = 12–14); vs. sham, ^@^*p* < 0.05, ^@@^*p* < 0.01, ^@@@^*p* < 0.001, ^@@@@^*p* < 0.0001; vs. MCAO/R, *p < 0.05, **p < 0.01, ***p < 0.001.

We investigated the effects of AOM on body weight, neurological deficit, spatial learning, and memory in transient cerebral ischemic rat model. The rats were subjected to 2 h of MCAO cerebral ischemia plus 14 days of reperfusion. AOM (6.3 g/kg per day) was orally administered to the rats for 13 days after surgical operation. As shown in Figure 4A, the body weight was time-dependently decreased in the MCAO vehicle group after surgical operation. AOM treatment group had significantly increased body weight than the MCAO vehicle group. Meanwhile, we evaluated the neurological dysfunction scores. The MCAO vehicle group had remarkably higher neurological deficit scores than sham control group. AOM treatment group showed a trend to reduce the neurological severity scores but had no statistically difference from the MCAO vehicle group (Figure 4B). We further evaluated spatial learning and long-term and short-term memory functions with Morris water maze and novel objective test. In the Morris water maze test, after 4 days of training, we tested the spatial learning and long-term memory functions by recording the time to reach the platform, the distance in target quadrant, and latency time to reach the platform. The AOM treatment group spent less time to reach the hidden platform than the MCAO vehicle group at day 4 (Figure 4C). In the probe trial, the AOM treatment group had remarkably longer swimming routes in target region than the MCAO vehicle treatment group (Figure 4D). Statistical analysis revealed that the AOM-treated rats had significantly improved parameters in the distance in target quadrant, latency time to reach the platform, and total distance in habituation. The AOM-treated rats had more distance and time in target region and spent less time to reaching platform region (Figure 4E). We also used open-field test to measure anxiety (Figures 4F,G) and novel objective recognition to detect short-term memory (Figure 4H). In the open-field test, the AOM treatment increased the distance and the time in the center region and the entry times to the center, suggesting the antianxiety effects. However, there was no significant difference in locomotor activity among the groups showing similar total distances in the whole regions. In the novel objective recognition test, the AOM treatment group showed better memory to the familiar objects and had more interest in exploring new objects than the MCAO vehicle group. Those results suggest that AOM could improve the quality of life, promote spatial learning/memory and recognition capacity, and reduce anxiety in the post–ischemic stroke rats.

### AOM Promotes Adult Hippocampal Neurogenesis in Post-MCAO Ischemic Rats *via* Inducing BDNF Pathway

We then investigated the neurogenic effects of AOM on inducing proliferation and differentiation in the hippocampus of the post-MCAO ischemic rats by using immunofluorescence and Western blot analysis. Ki67 is a biomarker for indexing the cells at proliferation stage, while BrdU is an analog of thymidine for marking newly generated cells. The dual-immunofluorescent staining of BrdU/Ki67 was used for detecting newly generated cells that were at proliferating stage and DAPI for nuclei identification. In addition, we performed the immunofluorescent experiments to identify newborn immature neurons and newborn mature neurons by using DCX NeuN, respectively. Dual-immunostaining imaging of BrdU/DCX and BrdU/NeuN in the postischemic brain regions was investigated. The results showed that AOM extract significantly increased the dual-positive staining populations of BrdU/Ki67, BrdU/DCX, and BrdU/NeuN in the DG and SVZ of the MCAO rats. Those results indicate that AOM could promote neurogenesis in hippocampus, SVZ, and striatum of post-MCAO ischemic rats (Supplementary Figures 2–5).

**FIGURE 5:**
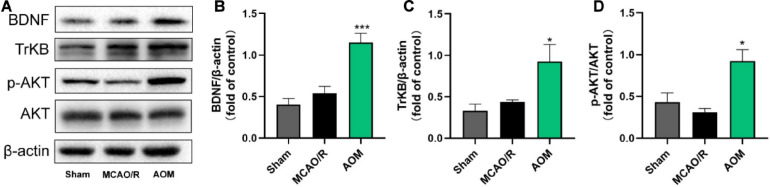
*Alpinia oxyphylla* Miq. up-regulated expression of BDNF, TrkB, and phosphorylated AKT in the hippocampus of transient MCAO cerebral ischemic rats. S.D. rats were divided into groups of sham, MCAO/R, and AOM. The rats were subjected to 2 h of MCAO cerebral ischemia plus 14 days of reperfusion. AOM extract (6.3 g/kg, dissolved in 5% ethanol and 5% PEG400) and vehicle solution were orally given to the rats at onset of reperfusion after 2 h of MCAO cerebral ischemia and daily administered for 13 days of reperfusion. **(A)** Representative immunoblot results for expression of BDNF, TrkB, and phosphorylated AKT. **(B)** Statistical analysis of relative expression level of BDNF in sham, MCAO/R, and AOM groups. **(C)** Statistical analysis of relative expression level of TrkB in sham, MCAO/R, and AOM groups. **(D)** Statistical analysis of relative expression level of phosphorylated AKT in sham, MCAO/R, and AOM groups. Data were presented as mean ± SEM (*n* = 6–8); vs. MCAO/R, *p < 0.05, ***p < 0.001.

Neurotrophic factor BDNF is a crucial cellular signaling regulator in the process of hippocampal neurogenesis for improving learning and memory functions (Egan et al., 2003). Functional TrkB signaling is a crucial player in the proliferation of NSCs and the survival and functional integration of newborn neurons in adult hippocampus (Bergami et al., 2008; Li et al., 2008). Thus, we detected whether AOM could regulate BDNF/TrkB/AKT signaling for improving neurogenesis in the hippocampus regions (Figure 5). There was no statistical difference in the expression of BDNF and p-AKT in the hippocampus regions between the sham control group and the MCAO vehicle group. The AOM treatment group had significantly higher expression levels of BDNF, TrkB, and p-AKT in the hippocampus regions than the MCAO vehicle group. Those results suggest that AOM could activate BDNF/TrkB/AKT signaling pathway and promote adult hippocampal neurogenesis in post-MCAO ischemic rats.

### *p*-Coumaric Acid Serves as a BDNF Inducer for Promoting Neurogenesis in NSCs

**FIGURE 6:**
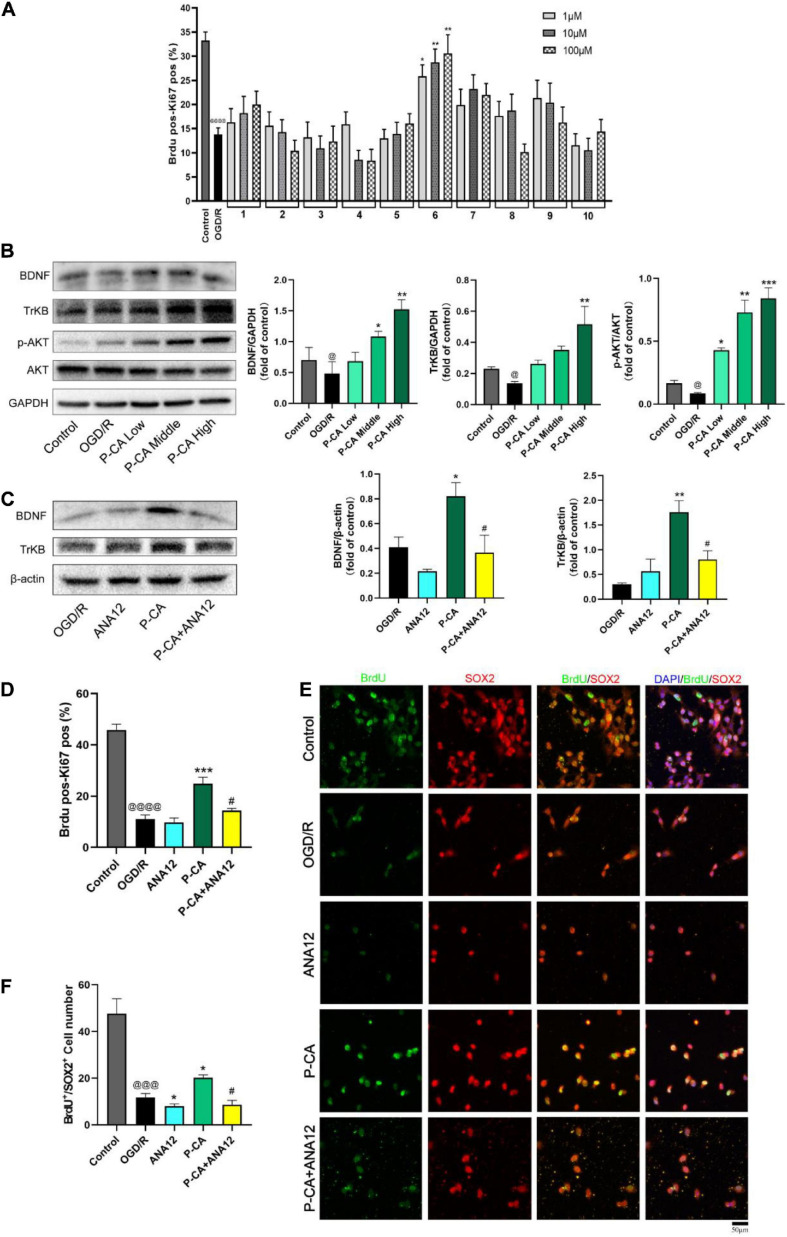
Identification of P-CA as an active compound from AOM with property of inducing BDNF/TrkB/AKT signaling and promoting NSC proliferation in cultured mouse cerebellum stem like C17.2 cells *in vitro*. To mimic cerebral ischemia–reperfusion injury *in vitro*, the C17.2 cells were subjected to 4 h of oxygen glucose deprivation plus 20 h of reoxygenation (OGD/R). By using high content screening (HCS) technology, we examined the effects of 10 active compounds from AOM (1, 10, and 100 μM) on the rates of BrdU/Ki67 dual-positive-staining cells and BDNF/TrkB/AKT signaling in the C17.2 cells under OGD/R exposure. **(A)** The effects of 10 compounds (1, 10 and 100 μM) on rates (%) of the BrdU and Ki67 dual positive staining cells detected by HCS technology. Ten compounds: 1. 5-Hydroxymethylfurfural; 2. Protocatechuic acid; 3. Catechin; 4. (-)-Epicatechol; 5. Protocatechuic aldehyde; 6. P-Coumaric acid; 7. Kaempferol; 8. Chrysin; 9. Nootkatone; 10. Tectochrysin. Effects of these compounds (1, 10, and 100 μM) on the rates of BrdU/Ki67 dual-positive-staining cells counted by HCS technology (right). Dual-positive staining of BrdU and Ki67 indicates the NSC proliferation-promoting activity. P-CA was found to be the most effective compound to induce proliferation of NSCs. **(B)** Western blot analysis indicating the effects of P-CA (1, 10, and 100 μM) on the expression of BDNF, TrkB, and p-Akt. P-CA dose-dependently up-regulated the expression of BDNF, TrkB, and p-Akt. **(C)** TrkB inhibitor ANA12 (20 μM) abolished the effects of P-CA (100 μM) on the expression of BDNF and TrkB. **(D)** The effects of P-CA (100 μM) on rates (%) of the BrdU and Ki67 dual-positive-staining cells detected by HCS technology. ANA12 (20 μM) abolished the effects of P-CA (100 μM) on the BrdU/Ki67 dual-positive-staining cells. **(E)** Representative immunofluorescent staining of BrdU (green) and SOX2 (red) with nucleus (blue). **(F)** Representative immunofluorescent imaging for BrdU (green) and SOX2 (red) positive-staining cells colocalized with nucleus (blue) in C17.2. ANA12 (20 μM) abolished the effects of P-CA (100 μM) on the BrdU/SOX2 dual-positive-staining cells. Data were presented as mean ± SD (*n* = 3); vs. control ^@^*p* < 0.05, ^@@@^*p* < 0.001, ^@@@@^*p* < 0.0001; vs. OGD/R *p < 0.05, **p < 0.01, ***p < 0.001; vs. P-CA ^#^*p* < 0.05.

We then screened the active compounds from AOM with the properties of inducing BDNF signaling and promoting neurogenesis in the cultured C17.2 cells exposed to 4-h OGD plus 20-h reoxygenation. Table 1 shows 10 compounds identified from AOM. The cells were treated with these compounds at the concentrations of 1, 10, and 100 μM. BrdU and Ki67 are commonly used biomarkers to analyze the proliferative activity of NSCs (Tanaka et al., 2011). By using HCS technology, we examined the rates of dual-positive staining of BrdU/Ki67 in the cells for identifying the proliferation-promoting effects. The compound 6, P-CA, was found to be the most effective ingredient to increase the BrdU/Ki67 dual-positive cells (Figure 6A). Meanwhile, P-CA treatment significantly up-regulated the expression of BDNF, TrkB, and p-AKT in the cells (Figure 6B). Cotreatment of ANA12 (20 μM, a BDNF/TrkB specific inhibitor) abolished the effects of P-CA (100 μM) on the induction of the expression of BDNF and TrKB (Figure 6C) and the increased rates of BrdU/Ki67 dual-positive staining in the C17.2 cells (Figure 6D). Furthermore, we performed immunofluorescent studies on the costaining of BrdU/SOX2, in which SOX2 is a critical marker for embryonic stem cells. P-CA treatment significantly increased the rates of BrdU/SOX2 positive cells whose effect was also abolished the cotreatment of ANA12 (Figures 6E,F). Those results suggest that P-CA could increase the survival of NSCs and stimulate the proliferation of NSCs *via* inducing BDNF/TrkB/AKT signaling pathway.

### P-CA Treatment Increases Body Weight, Improves Neurological Deficit Score, Promotes Spatial Learning and Memory, and Reduces Anxiety in Post-MCAO Ischemic Rats

**FIGURE 7:**
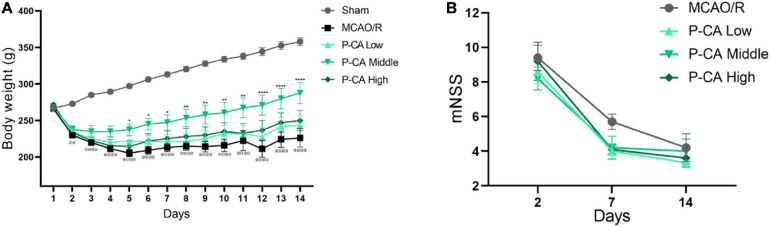
P-CA treatment increased body weight, improved spatial learning and memory capacity, and reduced anxiety in MCAO rats. S.D. rats were divided into sham control group (Sham), MCAO cerebral ischemia–reperfusion vehicle group (MCAO/R), and MCAO/R plus P-CA treatment groups (P-CA low 50 mg/kg, P-CA middle 100 mg/kg, P-CA high 200 mg/kg). The rats were subjected to 2 h of MCAO cerebral ischemia plus 14 days of reperfusion. P-CA 50, 100, and 200 mg/kg were intragastrically administered into the rats for following 13 consecutive days, whereas vehicle solution was used in sham-operated group and MCAO vehicle group. **(A)** Body weight changes: P-CA increased body weight of post-MCAO ischemic rats. **(B)** Neurological deficit scores (mNSS) assessed on the 2nd, 7th, and 14th day after MCAO surgery. The P-CA group had no effect on mNSS statistically when compared with MCAO/R group. Data were presented as mean ± SEM (*n* = 10); vs. sham control, ^@@^*p* < 0.001, ^@@@@^*p* < 0.0001; vs. MCAO/R, *p < 0.05, **p < 0.01, ****p < 0.0001.

**FIGURE 8:**
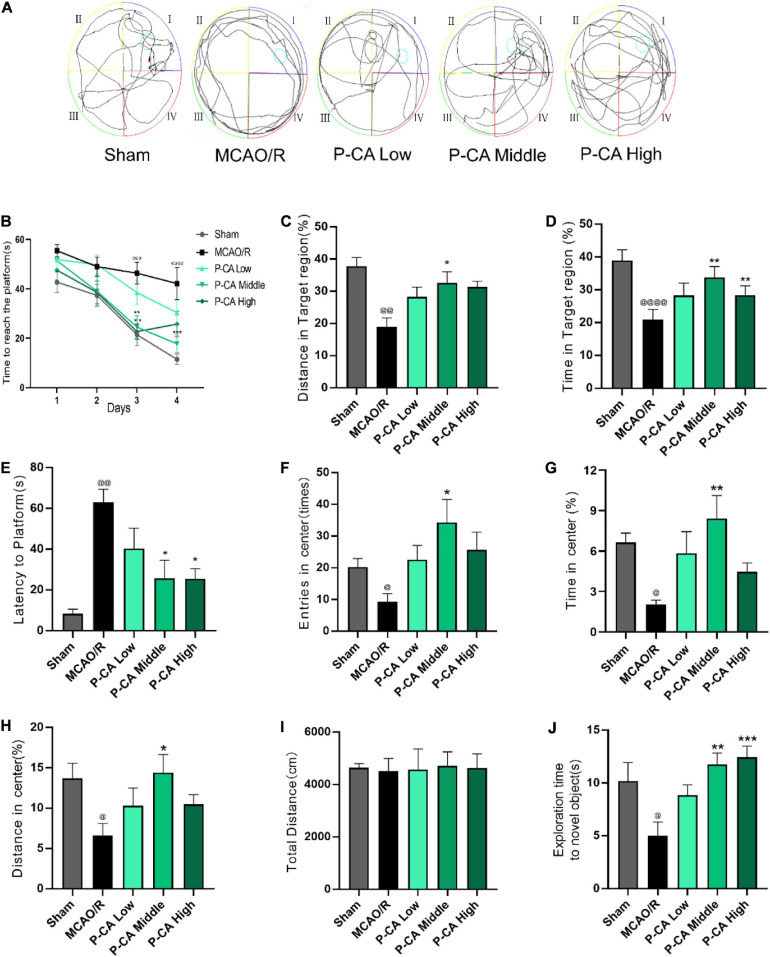
P-CA treatment increased body weight, improved spatial learning and memory capacity, and reduced anxiety in MCAO rats. S.D. rats were divided into sham control group (Sham), MCAO cerebral ischemia–reperfusion vehicle group (MCAO/R), and MCAO/R plus P-CA treatment groups (P-CA low 50 mg/kg, P-CA middle 100 mg/kg, P-CA high 200 mg/kg). The rats were subjected to 2 h of MCAO cerebral ischemia plus 14 days of reperfusion. P-CA 50, 100, and 200 mg/kg were intragastrically administered into the rats for following 13 consecutive days, whereas vehicle solution was used in sham-operated group and MCAO vehicle group. **(A–E)** Morris water maze tests for spatial learning and long-term memory in the sham control, MCAO/R, and P-CA treatment groups: P-CA treatment revealed to improve spatial learning and long-term memory. **(B)** Escape latency in 4 days of hidden platform tests. **(A)** Swimming routes in the probe trial. **(C)** Distance and **(D)** time in the target quadrant of the probe tail test, and **(E)** latency time before reaching platform region. **(F–H)** Open-field test for anxiety in the sham control, MCAO/R, and P-CA treatment groups: Distance, time and entries in center region were recorded. P-CA treatment reduced the anxiety but had no effect on total distance moved during habituation **(I)**. **(J)** Novel objective recognition test for short-term memory: P-CA increased exploration time on novel object in test phase of the MCAO rats. Data were presented as mean ± SEM (*n* = 10); vs. sham control, ^@^*p* < 0.01, ^@@^*p* < 0.001, ^@@@^*p* < 0.001, ^@@@@^*p* < 0.0001; vs. MCAO/R, *p < 0.05, **p < 0.01, ***p < 0.001.

We next investigated the effects of P-CA on body weight, mNSS, spatial learning, and memory in post-MCAO ischemic rats. In the P-CA treatment group, P-CA 50, 100, and 200 mg/kg were intragastrically administered into the rats at the onset of reperfusion after MCAO ischemia and used daily for 13 consecutive days. Vehicle solution was used in sham-operated group and MCAO vehicle group. The P-CA treatment increased body weight but had no effect on the neurological severity scores statistically (Figures 7A,B). We also performed the Morris water maze to evaluate the spatial learning and long-term memory functions (Figures 8A–E), the open-field test to measure anxiety (Figures 8F–I), and the novel objective recognition to detect short-term memory (Figure 8J). In the Morris water maze test, the P-CA treatment significantly increased the distance and time in the target quadrant of the probe trial and decreased the latency time to reach the platform, suggesting the improvement of spatial learning and long-term memory functions. In the open-field test, the P-CA treatment increased the distance and time in center and the entry times in center region, suggesting the effects of reducing anxiety. In the novel objective recognition test, P-CA treatment increased the exploration time on novel object in test phase, indicating the improvement of short memory. These results suggest that P-CA could promote spatial learning, increase short and long-term memory abilities, and reduce anxiety in post–ischemic stroke rats.

### P-CA Treatment Promotes Adult Hippocampal Neurogenesis in Post-MCAO Ischemic Rat Brains *via* Inducing BDNF Signaling Pathway

**FIGURE 9:**
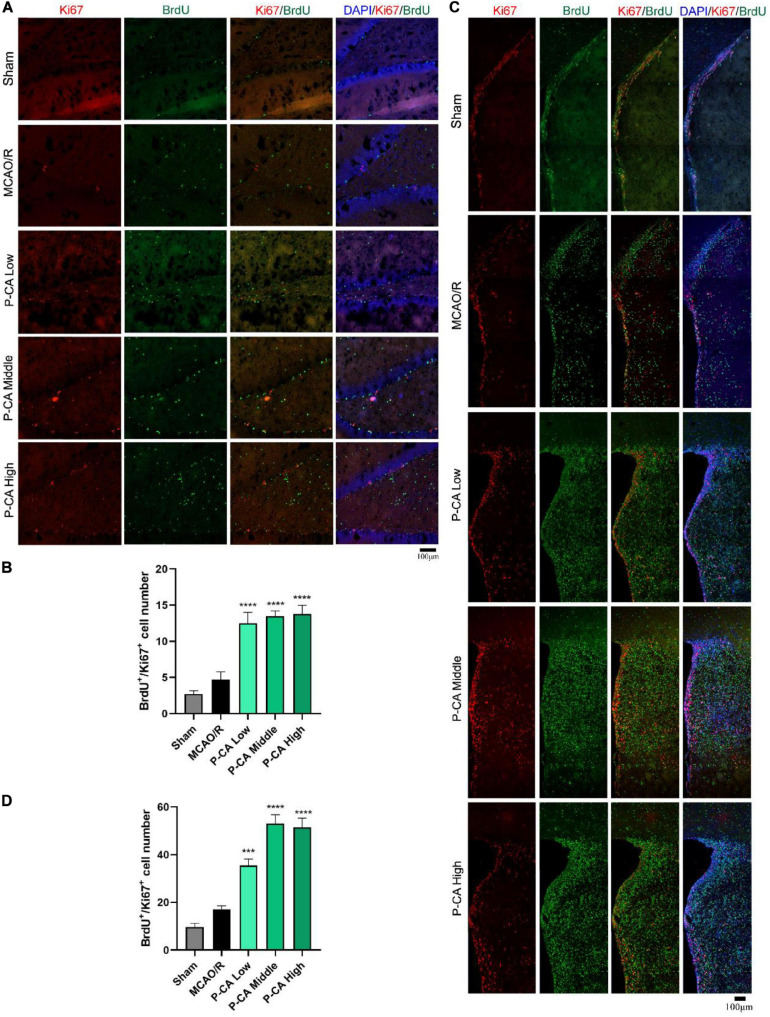
P-CA promoted proliferation in hippocampus, SVZ, and striatum of transient MCAO ischemic rats. S.D. rats were divided into sham, MCAO/R and MCAO/R plus P-CA treatment groups (P-CA low 50 mg/kg, P-CA middle 100 mg/kg, P-CA high 200 mg/kg). The rats were subjected to 2 h of MCAO cerebral ischemia plus 14 days of reperfusion. P-CA 50, 100, and 200 mg/kg were intragastrically administered into the rats for following 13 consecutive days, whereas vehicle solution was used in sham-operated group and MCAO vehicle group. **(A)** Representative immunofluorescent imaging for BrdU (green) and Ki67 (red) positive-staining cells colocalized with nucleus (blue) in hippocampal DG. Dual-positive staining of BrdU/Ki67 refers to the newly generated cells which are still in proliferating. **(B)** Statistical analysis of BrdU^+^/Ki67^+^ cell number in hippocampus in sham, MCAO/R, and P-CA groups. **(C)** Representative immunofluorescent imaging for BrdU (green) and Ki67 (red) positive-staining cells colocalized with nucleus (blue) in subventricular zone and striatum. **(D)** Statistical analysis of BrdU^+^/Ki67^+^ cell number in striatum and SVZ in sham, MCAO/R, and P-CA groups. Data were presented as mean ± SEM (*n* = 3-5 rats per group, 3 tissue sections per brain region used for imaging analysis); vs. MCAO/R, ***p < 0.001, ****p < 0.0001.

**FIGURE 10:**
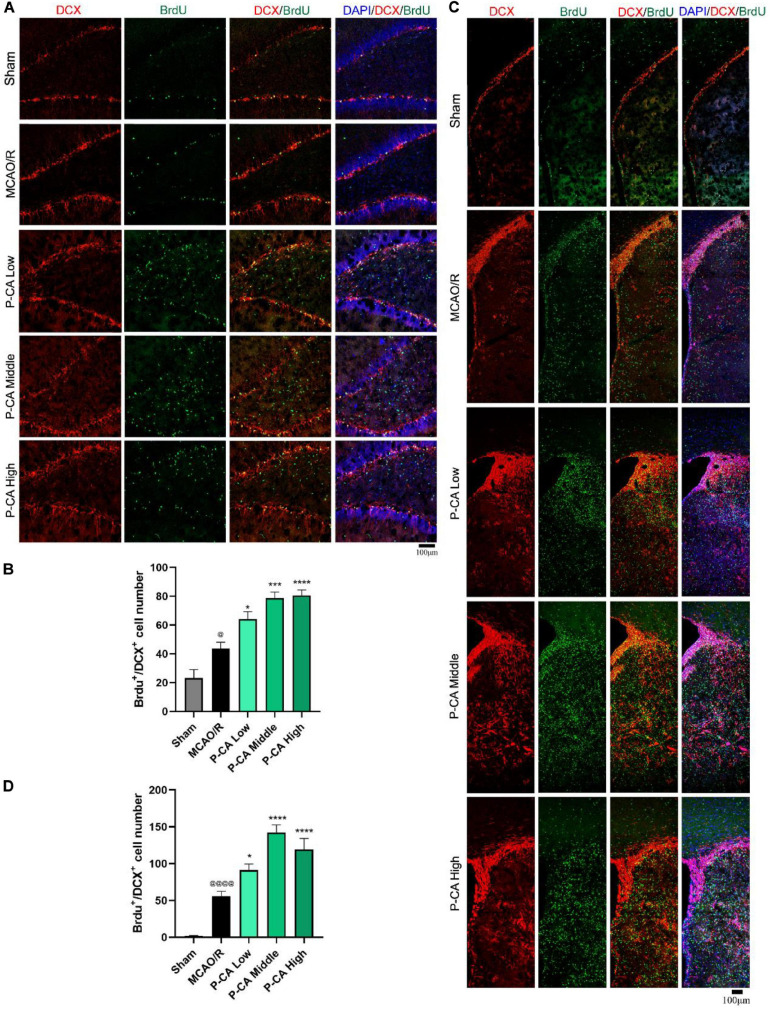
P-CA promoted NSC differentiation into immature neurons in hippocampus and striatum of transient MCAO ischemic rats. S.D. rats were divided into sham, MCAO/R and MCAO/R plus P-CA treatment groups (P-CA low 50 mg/kg, P-CA middle 100 mg/kg, P-CA high 200 mg/kg). The rats were subjected to 2 h of MCAO cerebral ischemia plus 14 days of reperfusion. P-CA 50, 100, and 200 mg/kg were intragastrically administered into the rats for following 13 consecutive days, whereas vehicle solution was used in sham-operated group and MCAO vehicle group. **(A)** Representative immunofluorescent imaging for BrdU (green) and DCX (red) positive-staining cells colocalized with nucleus (blue) in hippocampal dentate gyrus (DG). Dual-positive staining of DCX/BrdU refers to the newly differentiated immature neurons. **(B)** Statistical analysis of BrdU^+^/DCX^+^ cell number in hippocampus in sham, MCAO/R, and P-CA groups. **(C)** Representative immunofluorescent imaging for BrdU (green) and DCX (red) positive-staining cells colocalized with nucleus (blue) in subventricular zone and striatum. **(D)** Statistical analysis of BrdU^+^/DCX^+^ cell number in striatum in sham, MCAO/R, and P-CA groups. Data were presented as mean ± SEM (*n* = 3-5 rats per group, 3-4 tissue sections per brain region used for imaging analysis); vs. sham, ^@^*p* < 0.05, ^@@@@^*p* < 0.0001; vs. MCAO/R, *p < 0.05, ***p < 0.001, ****p < 0.0001.

**FIGURE 11:**
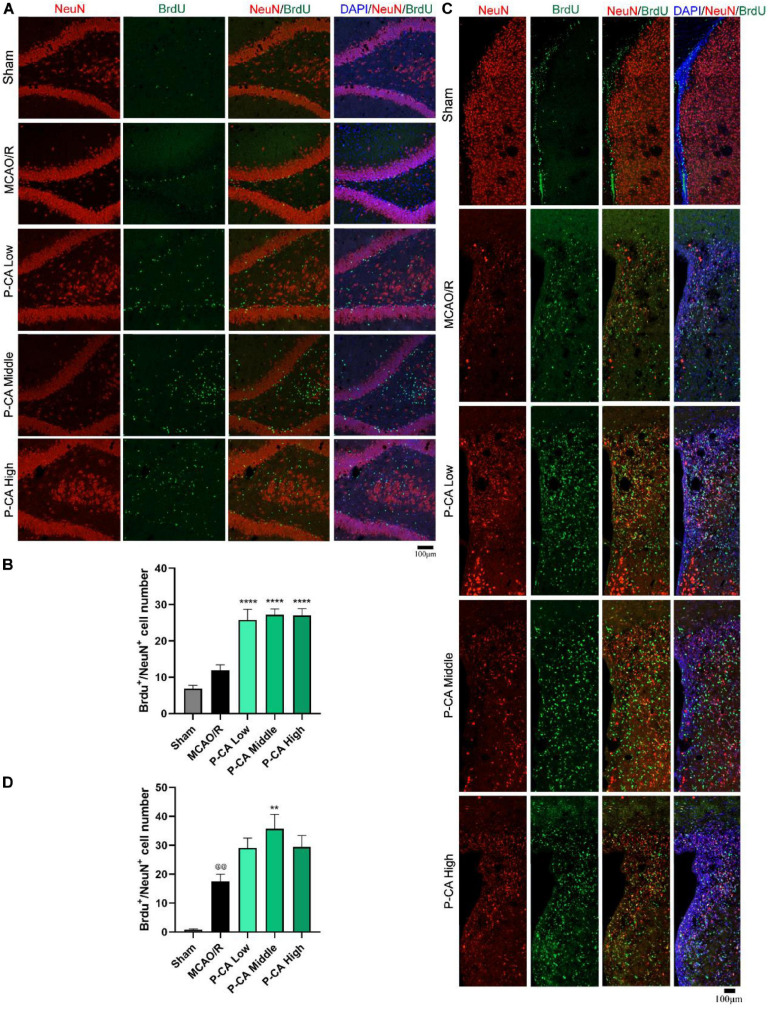
P-CA promoted differentiation into mature neurons in hippocampus and striatum of transient MCAO ischemic rats. S.D. rats were divided into sham, MCAO/R and MCAO/R plus P-CA treatment groups (P-CA low 50 mg/kg, P-CA middle 100 mg/kg, P-CA high 200 mg/kg). The rats were subjected to 2 h of MCAO cerebral ischemia plus 14 days of reperfusion. P-CA 50, 100, and 200 mg/kg were intragastrically administered into the rats for following 13 consecutive days, whereas vehicle solution was used in sham-operated group and MCAO vehicle group. **(A)** Representative immunofluorescent imaging for BrdU (green) and NeuN (red) positive-staining cells colocalized with nucleus (blue) in hippocampal dentate gyrus (DG). Dual-positive staining of DCX/BrdU refers to the newly formed mature neurons. **(B)** Statistical analysis of BrdU^+^/NeuN^+^ cell number in hippocampus in sham, MCAO/R, and P-CA groups. **(C)** Representative immunofluorescent imaging for BrdU (green) and NeuN (red) positive-staining cells colocalized with nucleus (blue) in subventricular zone and striatum. **(D)** Statistical analysis of BrdU^+^/NeuN^+^ cell number in striatum in sham, MCAO/R, and P-CA groups. Data were presented as mean ± SEM (*n* = 3–5 rats per group, three tissue sections per brain region used for imaging analysis). vs. sham control, ^@@^*p* < 0.01; vs. MCAO/R, **p < 0.01, ****p < 0.0001.

We then investigated the effects of P-CA on promoting hippocampal neurogenesis in the post-MCAO ischemic rats. We detected dual-positive staining of BrdU/Ki67 for cell proliferation in hippocampal DG, SVZ, and striatum regions where DAPI was used for nuclei identification (Figures 9A–D). The P-CA treatment remarkably increased the rates of the BrdU/Ki67–positive cells in the hippocampal DG, SVZ, and striatum regions of the postischemic brain regions. Furthermore, we identified newborn immature neurons and newborn mature neurons as detected with BrdU/DCX (Figures 10A–D) and BrdU/NeuN (Figures 11A–D), respectively. As expected, P-CA treatment promoted the production of newborn immature and mature neurons in the hippocampus and striatum of the postischemic brains. We also performed the imaging analysis at XYZ planes with the dual staining of BrdU/Ki67, BrdU/DCX, and BrdU/NeuN in the DG and SVZ. P-CA treatment increased the dual-positive staining of BrdU/Ki67, BrdU/DCX, and BrdU/NeuN in the DG and SVZ (Supplementary Figure 6). These results indicate that P-CA could improve NSC differentiation for neurogenesis in poststroke treatment.

**FIGURE 12:**
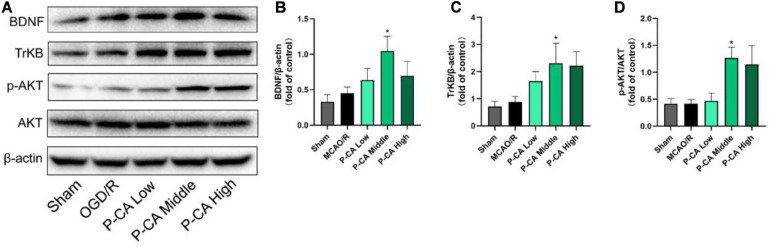
P-CA up-regulated expression of TrkB and phosphorylated AKT in the hippocampus of transient MCAO cerebral ischemic rats. S.D. rats were divided into sham control group (Sham), MCAO cerebral ischemia–reperfusion vehicle group (MCAO/R), and MCAO/R plus P-CA treatment groups (P-CA low 50 mg/kg, P-CA middle 100 mg/kg, P-CA high 200 mg/kg). The rats were subjected to 2 h of MCAO cerebral ischemia plus 14 days of reperfusion. P-CA 50, 100, and 200 mg/kg were intragastrically administered into the rats for following 13 consecutive days, whereas vehicle solution was used in sham-operated group and MCAO vehicle group. **(A)** Representative immunoblot results for expression of BDNF, TrkB, and phosphorylated AKT. **(B)** Statistical analysis of relative expression level of BDNF in sham, MCAO/R, and P-CA groups (50, 100, 200 mg/kg). **(C)** Statistical analysis of relative expression level of TrkB in sham, MCAO/R, and P-CA groups (50, 100, 200 mg/kg). **(D)** Statistical analysis of relative expression level of phosphorylated AKT in sham, MCAO/R, and P-CA groups (50, 100, 200 mg/kg). Data were presented as mean ± SEM (*n* = 6); vs. MCAO/R, *p < 0.05.

We further investigated the effects of P-CA on the expression of BDNF/TrkB/AKT signaling for improving neurogenesis in the hippocampus regions (Figure 12). There was no significant difference in the expression of BDNF, TrkB, and p-AKT in the hippocampus regions between sham control group and MCAO vehicle group. However, P-CA treatment significantly up-regulated the expression of BDNF, TrkB, and p-AKT in the hippocampus regions. Those results suggest that P-CA could activate BDNF/TrkB/AKT signaling pathway and promote adult hippocampal neurogenesis in post-MCAO ischemic rats.

## Discussion

*Alpinia oxyphylla* Miq. is a medicinal herb used for improving cognitive functions in TCM practice, but the scientific evidence of its use is lacking. In the present study, we demonstrate that AOM enhances adult hippocampal neurogenesis and improves cognitive impairment *via* inducing BDNF/TrkB/AKT signaling pathway in post–ischemic stroke treatment. Furthermore, we identify that P-CA is a representative active compound from AOM to activate BDNF/TrkB/AKT signaling pathway and induce adult hippocampal neurogenesis for enhancing cognitive functions. To our knowledge, this is the first report to demonstrate that AOM and its active compound P-CA could promote adult hippocampal neurogenesis and improve cognitive impairment *via* inducing BDNF/TrkB/AKT signaling pathway in postischemic brains.

Previous studies have reported the bioactivities of AOM in improving cognitive functions and antidepression (Koo et al., 2004; Shi et al., 2014; Yan et al., 2016). AOM improves cognitive functions in mouse cortex and hippocampus in amyloid β–induced AD mouse model (Shi et al., 2014). AOM exerts antidepressant-like effects through activating TrkB-mediated BDNF signal pathway (Yan et al., 2016). AOM enhances learning disability and protects CA1 hippocampal neurons in a mouse model with CCA occlusion (Koo et al., 2004; Wang et al., 2018). Herein, we tested the hypothesis that AOM could induce adult hippocampal neurogenesis and improve poststroke cognitive impairment *via* inducing BDNF/TrkB/AKT signaling pathway. AOM treatment significantly increased body weight, indicating the recovery from surgical injury and good quality of life in poststroke care. However, there was no statistical difference in the mNSS between the MCAO vehicle group and AOM treatment group. Importantly, Morris water maze and novel objective recognition test revealed that the AOM treatment significantly improved spatial learning/memory and enhanced cognitive functions in the post-MCAO ischemic rats with the impaired learning and memory functions. Meanwhile, AOM treatment enhanced cell proliferation and neuronal differentiation as marked by dual-immunofluorescent staining of BrdU/Ki67, BrdU/DCX, and BrdU/NeuN in hippocampal DG, SVZ, and striatum. Those results suggest that AOM could promote adult hippocampal neurogenesis, improve the spatial learning/memory impairment, and enhance cognitive functions in post-MCAO ischemic rats.

Brain-derived neurotrophic factor is a crucial neurotrophic factor to mediate hippocampal neurogenesis for improving learning and memory functions (Egan et al., 2003). BDNF not only promotes neurogenesis but also plays crucial roles in the survival and maintenance of neuronal cells (Wei et al., 2015). BDNF exerts its essential roles in promoting the hippocampal neurogenesis and rescuing cognitive and motor dysfunctions in a transgenic model of dementia (Goldberg et al., 2015). TrkB signaling is a crucial player in the proliferation of NSCs and the survival and functional integration of newborn neurons in adult hippocampus (Bergami et al., 2008; Li et al., 2008). In the present study, we found that AOM treatment significantly up-regulated the expression of BDNF, TrkB, and p-AKT in the hippocampus, indicating the induction of BDNF signaling pathway for neurogenesis in post-MCAO ischemic rats.

HCS technology is a sophisticated and efficient analytical method for drug discovery with application potentials for stem cell biology and genome studies (Fraietta and Gasparri, 2016). Combined HPLC and HCS technologies provide an effective platform for identifying the bioactive ingredients from medicinal herbal productions. With HCS technology, we examined the proliferation-promoting effects of the compounds identified from AOM extract. We counted the BrdU/Ki67 dual-positive populations in the C17.2 cells and found P-CA was the best compound to promote the proliferation of NSCs after the cells were exposed to 4-h OGD plus 20-h reoxygenation. SOX2 is a transcription factor expressed in the NSCs of the developing nervous system. SOX2 characterizes and marks undifferentiated precursor cells including stem cells for the early developmental stages of nerve system (Pevny and Nicolis, 2010). Fluorescent imaging studies revealed that P-CA treatment increased the dual-positive staining of BrdU/Ki67 and BrdU/SOX2 in the C17.2 cells after exposure to OGD. Therefore, P-CA could be a representative compound from AOM to promote the NSC proliferation. Furthermore, P-CA treatment dose-dependently up-regulated the expression of BDNF, TrkB, and p-AKT in the OGD-treated C17.2 cells, whose effects were blocked by ANA12, a BDNF/TrkB specific inhibitor. P-CA also increased the survival rates of the C17.2 cells. We then performed behavioral studies to evaluate the effects of P-CA on neurological deficit scores and spatial learning and memory functions in MCAO ischemic rats. P-CA treatment improved the spatial learning and long-term memory functions in the Morris water maze test, reduced anxiety in the open-field test, and increased short memory in novel objective recognition test. We then sought to determine the underlying mechanisms of P-CA in inducing hippocampal neurogenesis for improving cognitive functions. P-CA treatment significantly increased the dual-positive staining populations to BrdU/DCX and BrdU/NeuN in the DG, SVZ, and striatum of the postischemic brains, indicating the spontaneous neuronal differentiation. P-CA treatment also significantly increased the rates of dual-positive-staining cells to BrdU/Ki67, BrdU/DCX, and BrdU/NeuN in the hippocampal DG, SVZ, and striatum regions. Those data suggest that P-CA has the promoting effects on cell proliferation and neuronal differentiation. The promotion of the neurogenesis contributes to enhance spatial learning, increase short-term and long-term memory, and reduce anxiety in post–ischemic stroke rats.

*P*-coumaric acid, also named 4-hydroxycinnamic acid, is a phenolic acid. P-CA and its conjugates exhibit various bioactivities including antioxidant (Nasr et al., 2015), anti-inflammation (Pragasam et al., 2013), and anticancer (Shailasree et al., 2015). Previous studies reported that P-CA attenuated oxidative stress and reduced infarction size and neuronal vulnerability in cerebral ischemia–reperfusion injury (Guven et al., 2015; Sakamula and Thong-Asa, 2018). In the present study, the treatment of P-CA up-regulated the expression of BDNF, TrkB receptor, and p-AKT; promoted hippocampal neurogenesis; enhanced spatial learning; increased short and long-term memory; and attenuated anxiety in ischemic stroke rats. Furthermore, cotreatment of TrkB receptor inhibitor ANA12 blocked the effects of P-CA on proliferation and BDNF/TrkB/AKT signaling pathway in the NSCs. Thus, we conclude that P-CA could be a representative active compound contributing the neurogenesis-promoting effects of AOM through activating BDNF/TrkB/AKT signaling pathway. The enhanced hippocampal neurogenesis subsequently improves cognitive functions and reduces anxiety in the transient MCAO ischemic rats.

Nevertheless, with multiple ingredients in AOM, we should be cautious in interpreting the experimental results. In addition to P-CA, the synergic effects of the different ingredients might also contribute to the neurogenic effects of AOM, which remains to be further explored. Besides, we also performed molecular docking study to evaluate the capacity of P-CA to bind with BDNF. P-CA revealed to directly bind with BDNF (Supplementary Figure 7), suggesting the potential direct interaction between P-CA and BDNF for activating BDNF. However, how the binding regulation of P-CA up-regulates the expression of BDNF and affects the functions of BDNF remains to be further elucidated. Notably, TrkB antagonist, ANA12, abolished the effects of P-CA on the proliferation of NSCs, indicating that the neurogenic effects of P-CA should be BDNF-TrkB dependent. With complex biochemical and biophysical processes of the molecular dynamics and the intermolecular binding affinity, it is valuable to further explore the underlying mechanisms of P-CA to affect the BDNF protein and the neurogenesis in the postischemic brains.

## Conclusion

In conclusion, AOM could be an effective medicinal plant to promote adult hippocampal neurogenesis and improve cognitive impairment in post–ischemic stroke treatment. The underlying mechanisms could be related to inducing BDNF/TrkB/AKT signaling pathway. *p*-Coumaric acid, a representative active compound from AOM, could activate the BDNF/TrkB/AKT signaling pathway and mediate adult hippocampal neurogenesis, subsequently improving cognitive functions, and reducing anxiety.

## Data Availability Statement

The raw data suporting the conclusions of this article will be made available by the authors.

## Ethics Statement

The animal study was reviewed and approved by the University Committee on the Use of Live Animals in Teaching and Research, University of Hong Kong (CULAR No. 4664-18).

## Author Contributions

JS and YH designed the experiments, analyzed the data, and prepared the manuscript. YH and CP identified the compounds from AOM. YH, SC, and BT performed the cell and animal experiments and Western blot analysis. YH and SQ designed and performed the animal behavior study. ZW and BG performed the molecular docking simulation. JS, SQ, and BG interpreted the experimental data and discussed the experimental designs. CP provided fund for quality identification of AOM extract. JS received fund and organized all experimental activities and manuscript preparation. All authors contributed to the article and approved the submitted version.

## Conflict of Interest

The authors declare that the research was conducted in the absence of any commercial or financial relationships that could be construed as a potential conflict of interest.
