# Supplementary material for: Alpinia oxyphylla Miq. and Its Active Compound P-Coumaric Acid Promote Brain-Derived Neurotrophic Factor Signaling for Inducing Hippocampal Neurogenesis and Improving Post-cerebral Ischemic Spatial Cognitive Functions
Source: Front Cell Dev Biol. 2021 Jan 18;8:577790. doi: 10.3389/fcell.2020.577790 (PMC7849625; doi:10.3389/fcell.2020.577790)
Supplement: Supplementary file 1 [file Data_Sheet_1.pdf]

Supplementary Materials

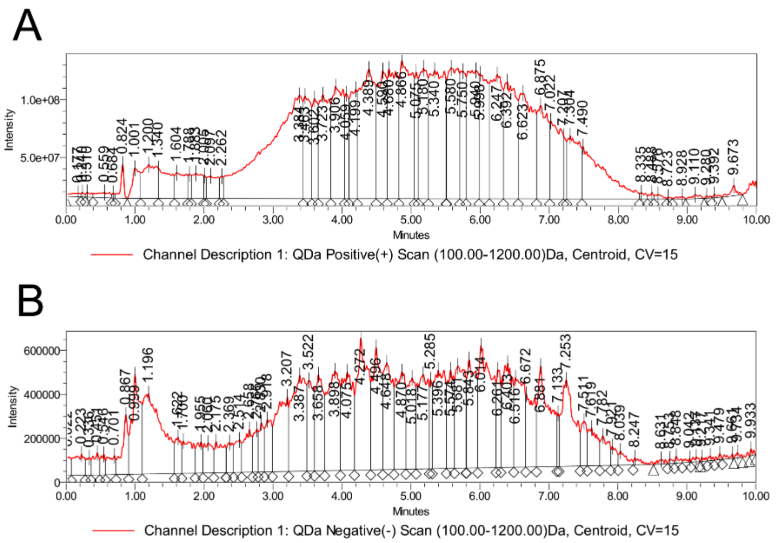

Supplemental Figure 1. ESI-MS chromatograms of AOM extract. LC-MS was operated on waters LC/MS ACQUITY QDA with CORTECS C18 column (4.6×50mm, 2.7μm), measured under condition of ionization method-ESI (+ -) and scanned range from 100 to 1000. **(A)** QDa positive scan of AOM extract **(B)** QDa negative scan of AOM extract

Supplemental Table 1 Representative compounds in AOM extract confirmed by LC-MS

Peak No.	Stander ESI	Sample ESI	Molecular weigh	Formula	Identification
1	$[M+H]^{+}$: 126.92	$[M+H]^{+}$: 126.91	126.11	$C_6H_6O_3$	5-Hydroxymethylfurfural
2	$[M-H]^{-}$: 153.05	$[M+H]^{+}$: 154.17	154.12	$C_7H_6O_4$	Protocatechuic acid
3	$[M+H]^{+}$: 290.95	$[M+H]^{+}$: 290.95	290.27	$C_{15}H_{14}O_6$	Catechin
4	$[M+H]^{+}$: 290.91	$[M+H]^{+}$: 290.94	290.26	$C_{15}H_{14}O_6$	(-)-Epicatechol
5	$[M+H]^{+}$: 138.90	$[M+H]^{+}$: 138.92	138.12	$C_7H_6O_3$	Protocatechuic aldehyde
6	$[M-H]^{-}$: 163.07	$[M+H]^{+}$: 164.77	164.16	$C_9H_8O_3$	P-Coumaric acid
7	$[M+H]^{+}$: 286.92	$[M+H]^{+}$: 286.92	286.23	$C_{15}H_{10}O_6$	Kaempferol
8	$[M+H]^{+}$: 254.94	$[M+H]^{+}$: 254.96	254.24	$C_{15}H_{10}O_4$	Chrysin
9	$[M+H]^{+}$: 219.06	$[M+H]^{+}$: 219.06	218.34	$C_{15}H_{22}O$	Nootkatone
10	$[M+H]^{+}$: 268.95	$[M+H]^{+}$: 268.95	268.26	$C_{16}H_{12}O_4$	Tectochrysin

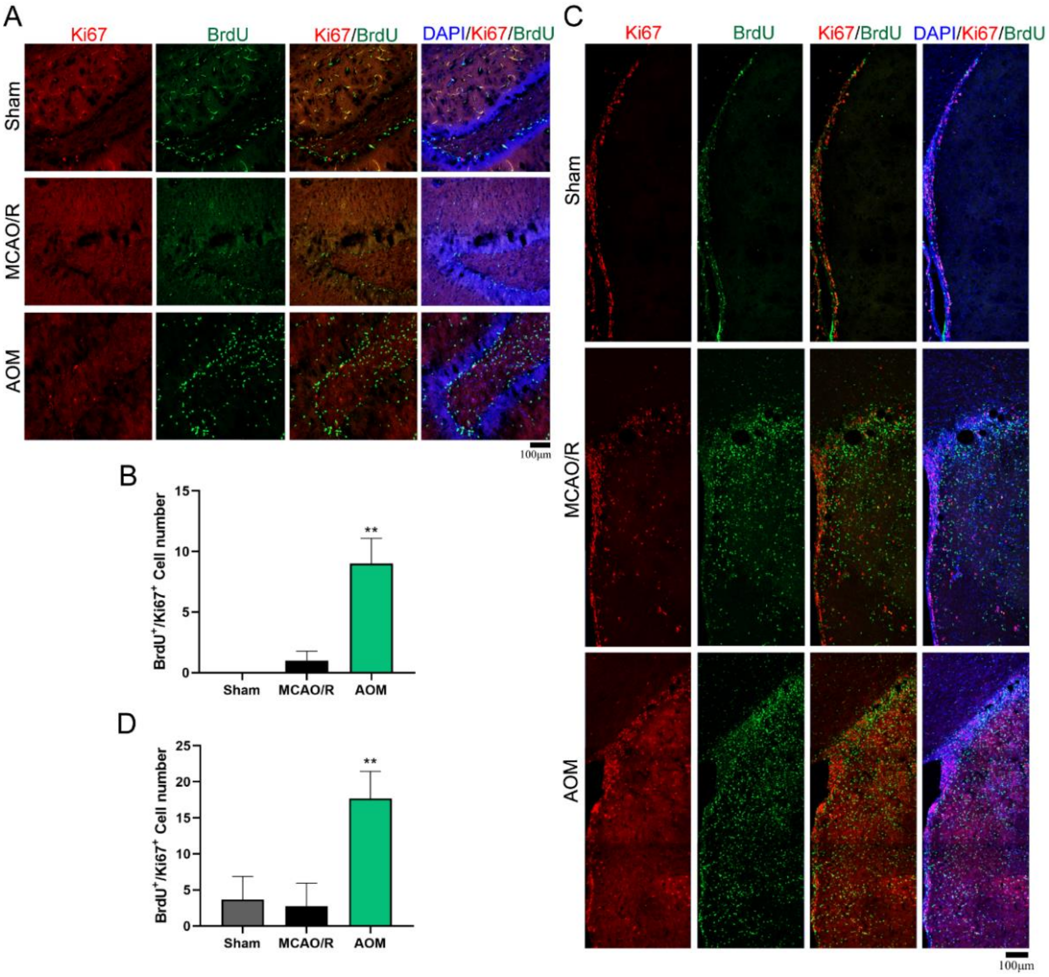

Supplemental Figure 2. AOM promoted proliferation in hippocampus and striatum of transient MCAO ischemic rats. S.D. rats were divided into groups of Sham, MCAO/R and AOM. The rats were subjected to 2 hours of MCAO cerebral ischemia plus 14 days of reperfusion. AOM extract (6.3g/kg, dissolved in 5% ethanol and 5% PEG400) and vehicle solution were orally given to the rats at onset of reperfusion after 2 hours of MCAO cerebral ischemia and daily administrated for 13 days of reperfusion. (**A**) Representative immunofluorescent imaging for BrdU (green) and Ki67 (red) positive staining cells co-localized with nucleus (blue) in hippocampal DG. Dual positive staining of BrdU/Ki67 refers to the newly generated cells which are still in proliferating. (**B**) Statistical analysis of $BrdU^{+}/Ki67^{+}$ cell number in hippocampus in Sham, MCAO/R and AOM groups. (**C**) Representative immunofluorescent imaging for BrdU (green) and Ki67(red) positive staining cells co-localized with nucleus (blue) in subventricular zone and striatum. (**D**) Statistical analysis of $BrdU^{+}/Ki67^{+}$ cell number in striatum and SVZ in Sham, MCAO/R and AOM groups. Data were presented as Mean ± SD (n=3-5 rats per group). Vs MCAO/R, ***p* < 0.01.

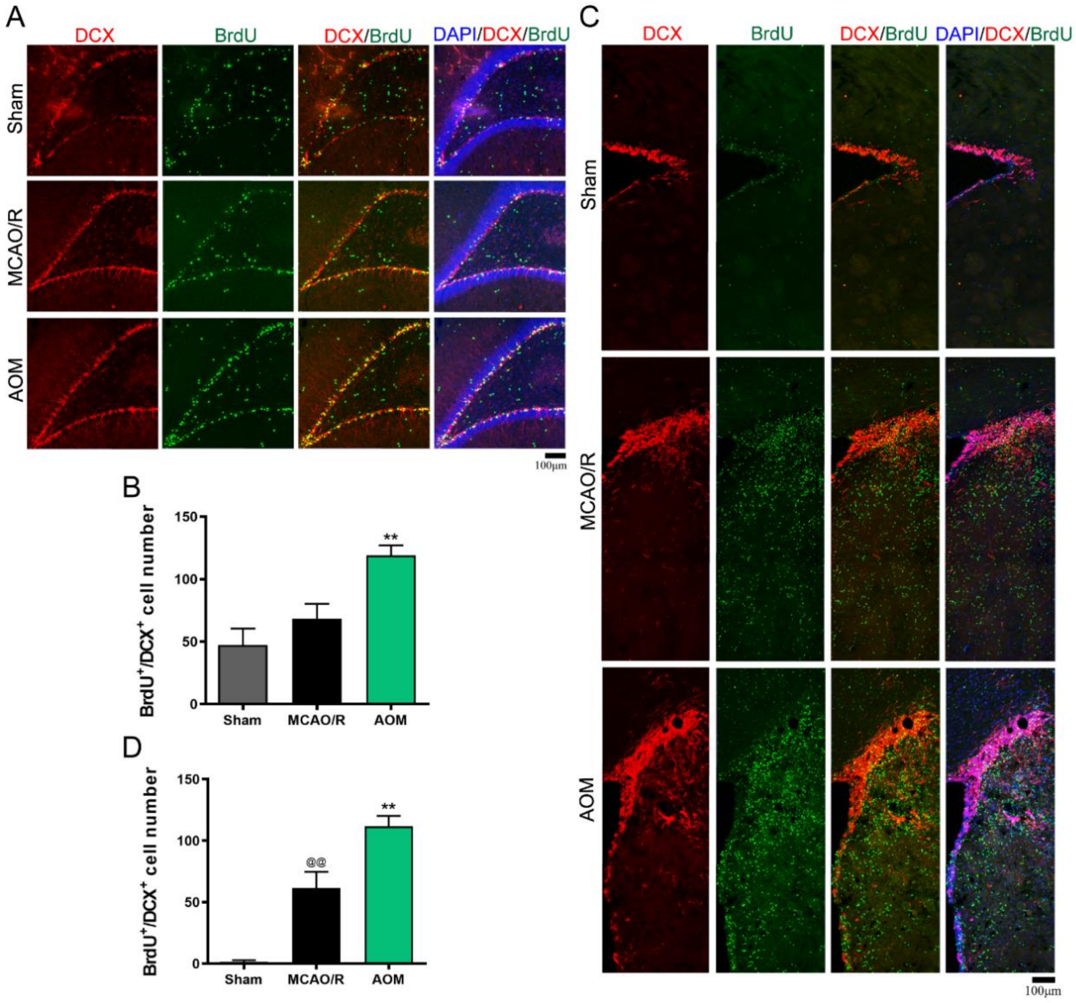

Supplemental Figure 3. AOM promoted neuronal differentiation in hippocampus and striatum of transient MCAO ischemic rats. S.D. rats were divided into groups of Sham, MCAO/R and AOM. The rats were subjected to 2 hours of MCAO cerebral ischemia plus 14 days of reperfusion. AOM extract (6.3g/kg, dissolved in 5% ethanol and 5% PEG400) and vehicle solution were orally given to the rats at onset of reperfusion after 2 hours of MCAO cerebral ischemia and daily administrated for 13 days of reperfusion. (**A**) Representative immunofluorescent imaging for BrdU (green) and DCX (red) positive staining cells co-localized with nucleus (blue) in hippocampal dentate gyrus (DG). Dual positive staining of DCX/BrdU refers to the newly differentiated immature neurons. (**B**) Statistical analysis of $BrdU^{+}/DCX^{+}$ cell number in hippocampus in Sham, MCAO/R and AOM groups. (**C**) Representative immunofluorescent imaging for BrdU (green) and DCX (red) positive staining cells co-localized with nucleus (blue) in subventricular zone and striatum. (**D**) Statistical analysis of $BrdU^{+}/DCX^{+}$ cell number in striatum and SVZ in Sham, MCAO/R and AOM groups. Data were presented as Mean ± SD (n=5-8 rats per group). Vs. Control $^{@@}p < 0.0$; Vs MCAO/R, $^{**}p < 0.01$.

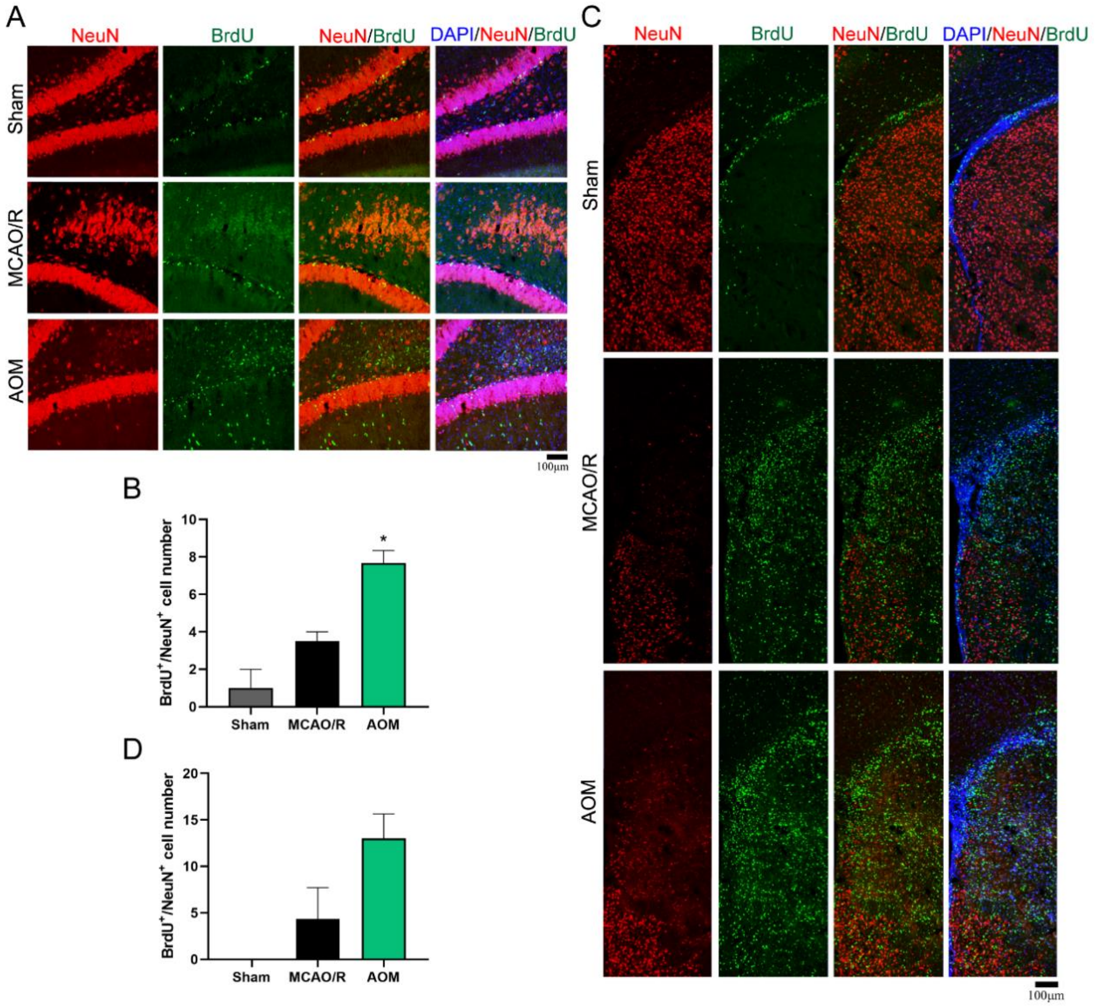

Supplemental Figure 4. AOM promoted neuronal differentiation in hippocampus and striatum of transient MCAO ischemic rats. S.D. rats were divided into groups of Sham, MCAO/R and AOM. The rats were subjected to 2 hours of MCAO cerebral ischemia plus 14 days of reperfusion. AOM extract (6.3g/kg, dissolved in 5% ethanol and 5% PEG400) and vehicle solution were orally given to the rats at onset of reperfusion after 2 hours of MCAO cerebral ischemia and daily administrated for 13 days of reperfusion.(**A**) Representative immunofluorescent imaging for BrdU (green) and NeuN (red) positive staining cells co-localized with nucleus (blue) in hippocampal dentate gyrus (DG). Dual positive staining of BrdU/NeuN refers to the newly formed mature neurons. (**B**) Statistical analysis of $BrdU^+/NeuN^+$ cell number in hippocampus in Sham, MCAO/R and AOM groups. (**C**) Representative immunofluorescent imaging for BrdU (green) and NeuN (red) positive staining cells co-localized with nucleus (blue) in subventricular zone and striatum. (**D**) Statistical analysis of $BrdU^+/NeuN^+$ cell number in striatum in Sham, MCAO/R and AOM groups. Data were presented as Mean ± SD (n=3-5 rats per group). Vs MCAO/R, *$p < 0.05$.

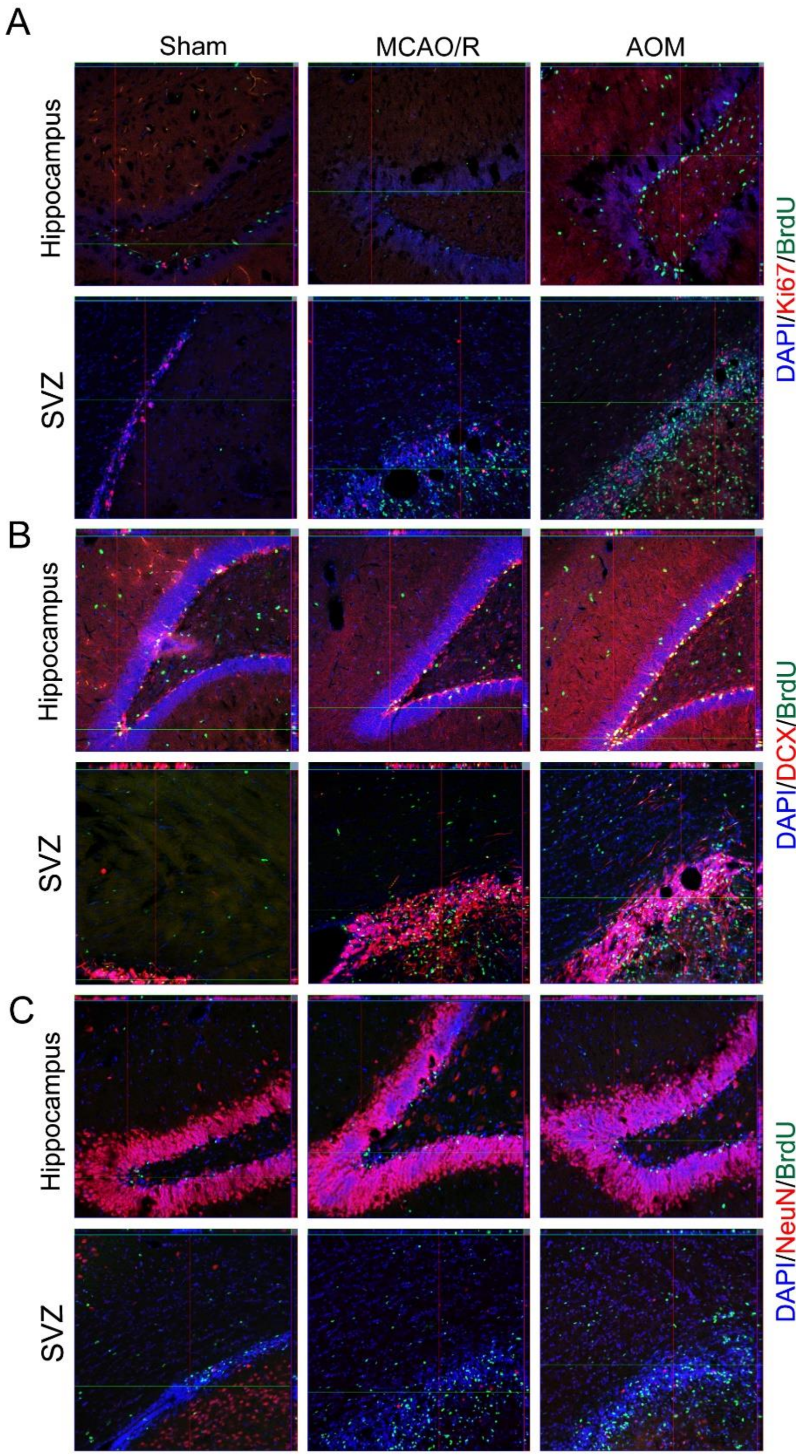

Supplemental Figure 5. Representative immunofluorescent imaging with XYZ planes of Sham, MCAO/R, AOM treatment groups.

(A) Representative immunofluorescent imaging for BrdU (green) and Ki67 (red) positive staining cells co-localized with nucleus (blue) in DG and SVZ. (B) Representative immunofluorescent imaging for BrdU (green) and DCX (red) positive staining cells co-localized with nucleus (blue) in DG and SVZ. (C) Representative immunofluorescent imaging for BrdU (green) and NeuN (red) positive staining cells co-localized with nucleus (blue) in DG and SVZ.

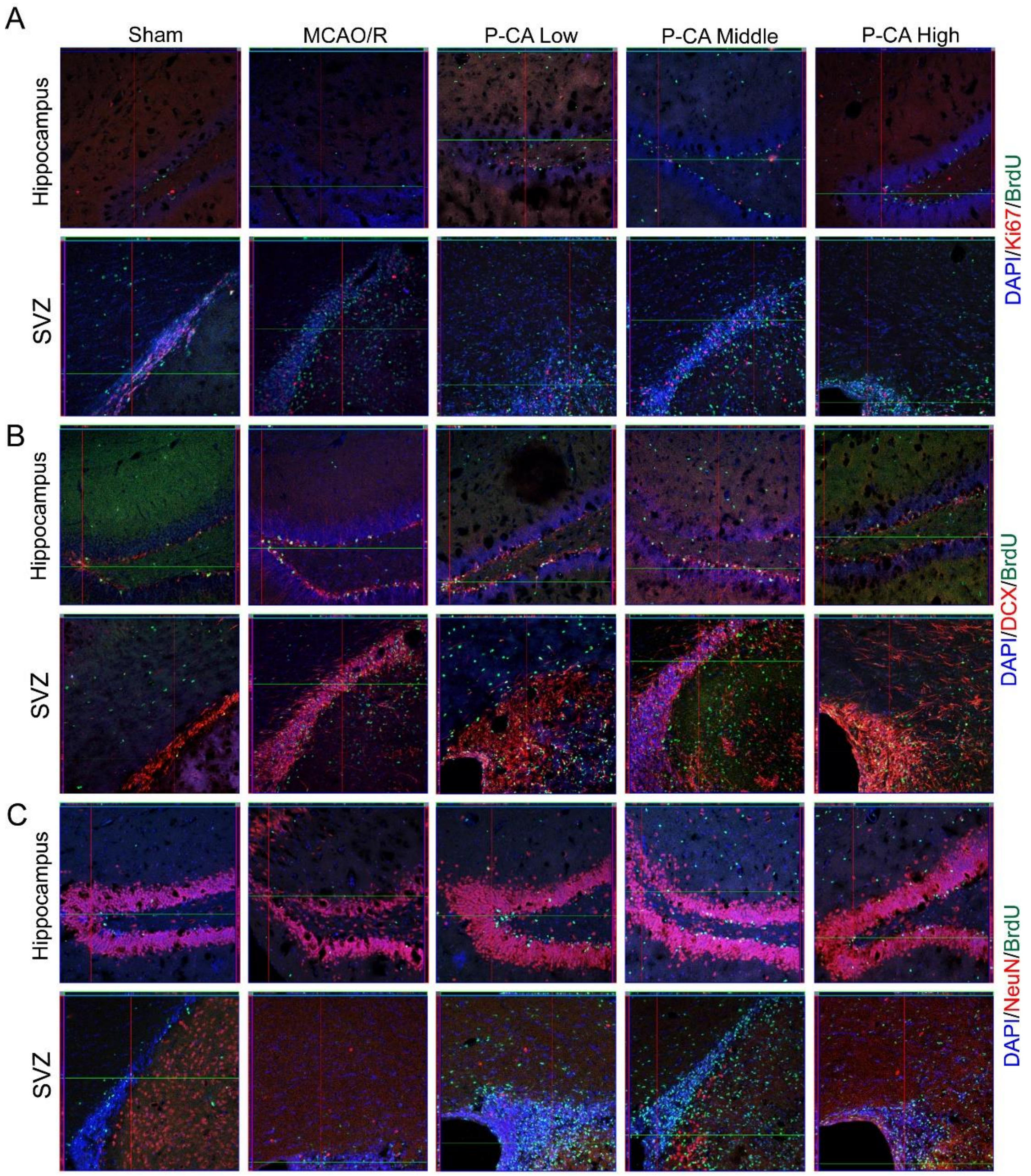

Supplemental Figure 6. Representative immunofluorescent imaging with XYZ planes of Sham, MCAO/R, P-CA treatment groups.

(A) Representative immunofluorescent imaging for BrdU (green) and Ki67 (red) positive staining cells co-localized with nucleus (blue) in DG and SVZ. (B) Representative immunofluorescent imaging for BrdU (green) and DCX (red) positive staining cells co-localized with nucleus (blue) in DG and SVZ. (C) Representative immunofluorescent imaging for BrdU (green) and NeuN (red) positive staining cells co-localized with nucleus (blue) in DG and SVZ.

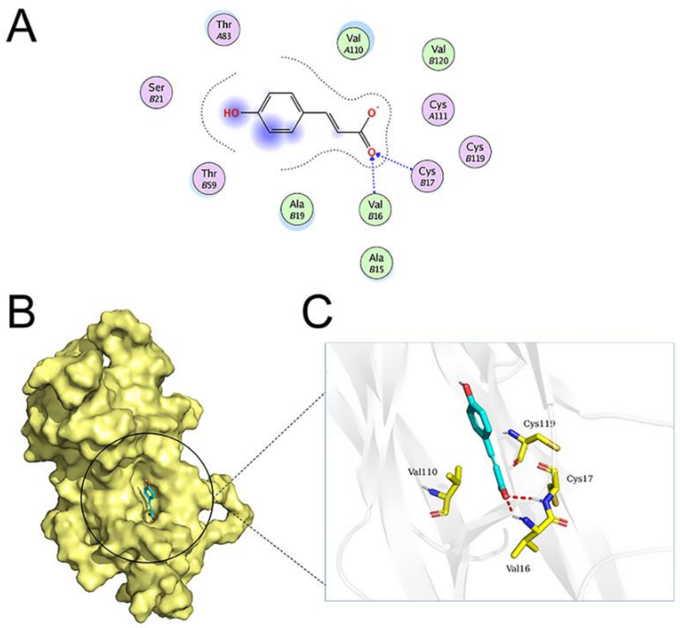

Supplemental Figure 7. Molecular docking simulation for binding modes of P-Coumaric acid and BDNF. (**A**) The 2D binding mode of P-Coumaric acid and BDNF. (**B**) The binding model of P-Coumaric acid on molecular surface of BDNF. P-Coumaric acid was colored in cyan and the molecular surface of BDNF was colored in pale yellow. (**C**) The 3D binding mode of P-Coumaric acid and BDNF. P-Coumaric acid was colored in cyan and the surrounding residues in the binding pockets are colored in yellow, the backbone of the receptor was depicted as white cartoon with transparency. The molecular docking scores of P-Coumaric acid with protein BDNF was -4.61 kcal/mol.

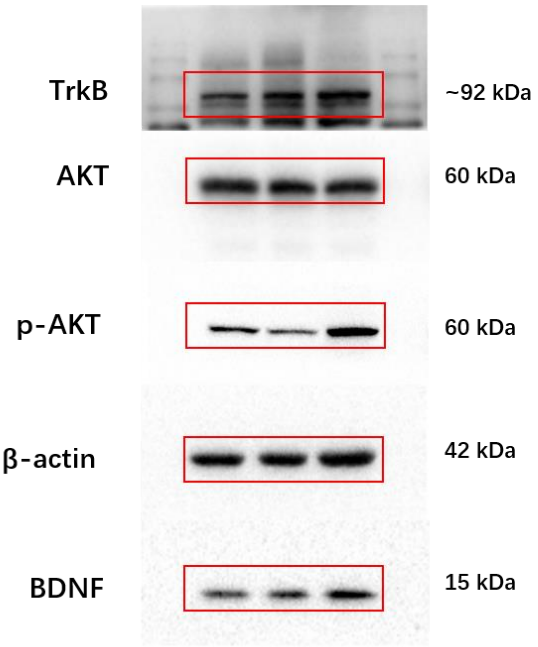

Supplemental Figure 8. Raw immunoblot results for expression of BDNF, TrkB, and phosphorylated AKT showed in **Figure 5**.

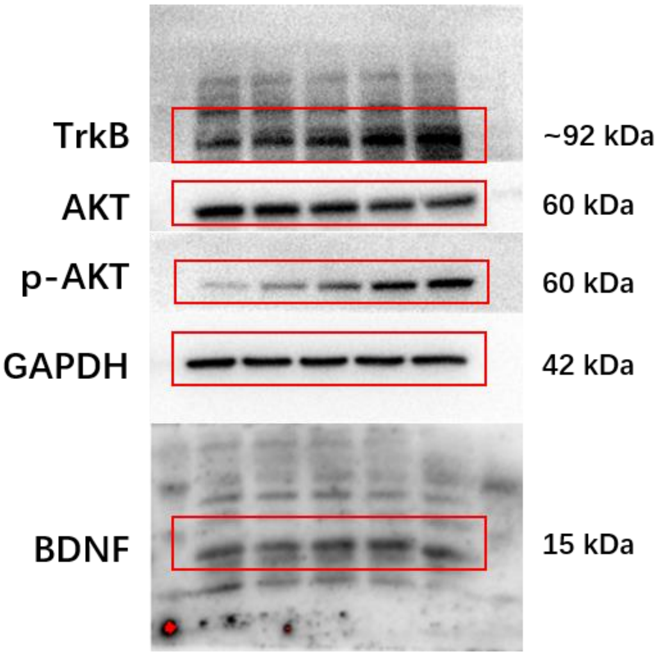

Supplemental Figure 9. Raw immunoblot results for expression of BDNF, TrkB, and phosphorylated AKT showed in **Figure 6B**.

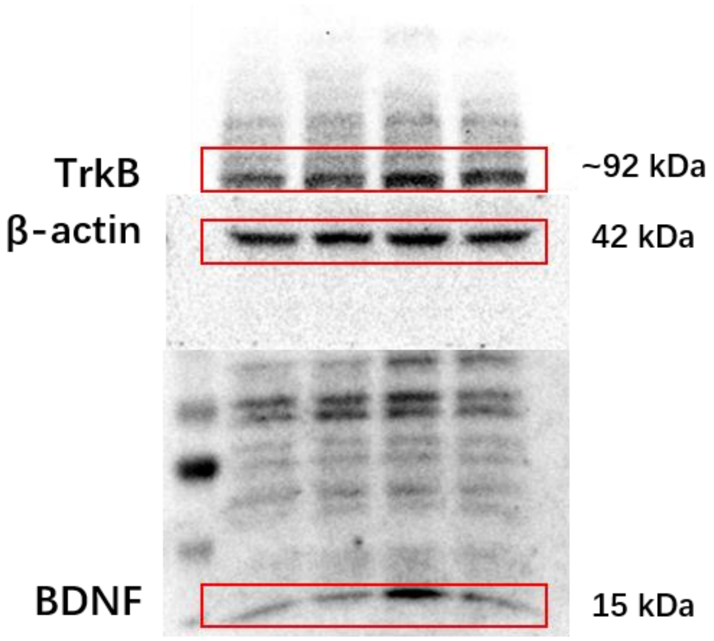

Supplemental Figure 10. Raw immunoblot results for expression of BDNF and TrkB showed in **Figure 6C**.

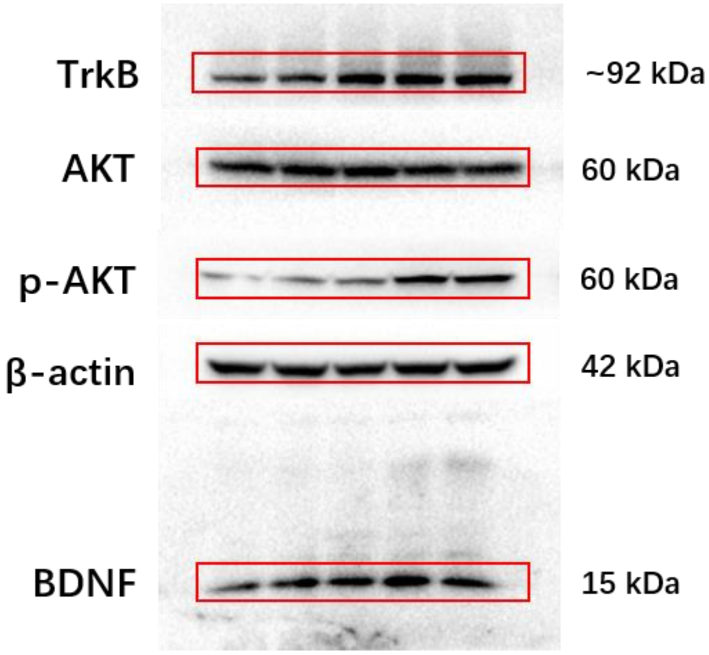

Supplemental Figure 11. Raw immunoblot results for expression of BDNF, TrkB, and phosphorylated AKT showed in **Figure 12**.
